# Orally bioavailable CDK9/2 inhibitor shows mechanism-based therapeutic potential in MYCN-driven neuroblastoma

**DOI:** 10.1172/JCI134132

**Published:** 2020-10-05

**Authors:** Evon Poon, Tong Liang, Yann Jamin, Susanne Walz, Colin Kwok, Anne Hakkert, Karen Barker, Zuzanna Urban, Khin Thway, Rhamy Zeid, Albert Hallsworth, Gary Box, Marli E. Ebus, Marco P. Licciardello, Yordan Sbirkov, Glori Lazaro, Elizabeth Calton, Barbara M. Costa, Melanie Valenti, Alexis De Haven Brandon, Hannah Webber, Nicolas Tardif, Gilberto S. Almeida, Rossitza Christova, Gunther Boysen, Mark W. Richards, Giuseppe Barone, Anthony Ford, Richard Bayliss, Paul A. Clarke, Johann De Bono, Nathanael S. Gray, Julian Blagg, Simon P. Robinson, Suzanne A. Eccles, Daniella Zheleva, James E. Bradner, Jan Molenaar, Igor Vivanco, Martin Eilers, Paul Workman, Charles Y. Lin, Louis Chesler

**Affiliations:** 1 Division of Clinical Studies and; 2 Division of Cancer Therapeutics, Institute of Cancer Research (ICR), London and Royal Marsden NHS Trust, Sutton, United Kingdom.; 3 Department of Molecular and Human Genetics, Baylor College of Medicine, Houston, Texas, USA.; 4 Division of Radiotherapy and Imaging, ICR, London, United Kingdom.; 5 Core Unit Bioinformatics, Comprehensive Cancer Center Mainfranken and Theodor Boveri Institute, Biocenter, University of Wurzburg, Wurzburg, Germany.; 6 Division of Molecular Pathology, ICR, London, and Royal Marsden NHS Trust, Sutton, United Kingdom.; 7 Department of Medical Oncology, Dana-Farber Cancer Institute, Boston, Massachusetts, USA.; 8 Cancer Research UK, Cancer Therapeutics Unit, ICR, London, United Kingdom.; 9 Prinses Maxima Center for Pediatric Oncology, Utrecht, Netherlands.; 10 School of Molecular and Cellular Biology, Faculty of Biological Sciences, University of Leeds, Leeds, United Kingdom.; 11 Department of Cancer Biology, Dana-Farber Cancer Institute, Boston, Massachusetts, USA.; 12 Department of Biological Chemistry and Molecular Pharmacology, Harvard Medical School, Boston, Massachusetts, USA.; 13 Cyclacel Ltd., Dundee, United Kingdom.; 14 Department of Medicine, Harvard Medical School, Boston, Massachusetts, USA.; 15 Comprehensive Cancer Center Mainfranken and Theodor Boveri Institute, Biocenter, University of Wurzburg, Wurzburg, Germany.

**Keywords:** Oncology, Cancer, Transcription, Translation

## Abstract

The undruggable nature of oncogenic Myc transcription factors poses a therapeutic challenge in neuroblastoma, a pediatric cancer in which *MYCN* amplification is strongly associated with unfavorable outcome. Here, we show that CYC065 (fadraciclib), a clinical inhibitor of CDK9 and CDK2, selectively targeted *MYCN*-amplified neuroblastoma via multiple mechanisms. CDK9 — a component of the transcription elongation complex P-TEFb — bound to the *MYCN*-amplicon superenhancer, and its inhibition resulted in selective loss of nascent *MYCN* transcription. MYCN loss led to growth arrest, sensitizing cells for apoptosis following CDK2 inhibition. In *MYCN*-amplified neuroblastoma, MYCN invaded active enhancers, driving a transcriptionally encoded adrenergic gene expression program that was selectively reversed by CYC065. MYCN overexpression in mesenchymal neuroblastoma was sufficient to induce adrenergic identity and sensitize cells to CYC065. CYC065, used together with temozolomide, a reference therapy for relapsed neuroblastoma, caused long-term suppression of neuroblastoma growth in vivo, highlighting the clinical potential of CDK9/2 inhibition in the treatment of *MYCN*-amplified neuroblastoma.

## Introduction

The prominent role of Myc family protooncogene transcription factors (TFs) (*MYC*, *MYCN*, *MYCL*) in the genesis of adult and childhood cancers makes these TFs attractive targets for drug discovery and development (1). However, the intrinsically disordered structure of Myc proteins and an incomplete understanding of how Myc, a master regulator of the transcriptomic and epigenetic landscape, coopts oncogenesis to drive cellular transformation are 2 major factors that impede traditional drug discovery approaches (1).

Across many cancers, a singular feature of oncogenic Myc activity is an increase in the abundance of its full-length protein (2). This suggests that Myc protein dosage itself is transforming. Strategies to attenuate Myc levels may be sufficient for achieving a therapeutic index in tumors versus normal tissues by selectively targeting oncogenic programs rather than tissue maintenance programs where Myc regulates homeostatic ribosome biogenesis and cellular proliferation (3, 4). In both normal and tumor cells, Myc functions primarily as an activator of transcription. When bound to DNA, Myc increases proximal chromatin accessibility via recruitment of histone acetyltransferases (5) and drives transcription elongation through recruitment of the elongation factor P-TEFb (cyclinT1:CDK9) (6) and RNA polymerase II–associated (RNAPII-associated) topoisomerases (7). A consensus has emerged that, when deregulated, Myc proteins act as selective amplifiers of gene expression (8, 9). Although Myc deregulation leads to a global increase in cellular mRNA in an oncogenic context, transformation driven by Myc in neuroblastoma (NB), a developmental tumor of neural crest origin, is associated with selective (and enhancer dependent) upregulation of a limited set of lineage-related genes, expression of which normally constitutes a transcriptomic core regulatory circuit (CRC) that underlies neural identity and deregulation of which drives growth and proliferation of these tumors (10–12).

We and others have found that MYCN globally upregulates and reshapes the NB gene expression landscape through the invasion of tissue-specific active enhancers that establish NB identity (13). In particular, large superenhancers that are adjacent to several TFs that demarcate the recently described adrenergic state of NB exhibit strong MYCN binding and are selectively regulated by MYCN (13). Expression of these TFs, including *GATA3*, *PHOX2A*, *PHOX2B*, *HAND2/TWIST1*, *TBX2*, and *ISL1*, is essential in *MYCN*-amplified NB (11, 13), suggesting that an oncogenic feed-forward interaction among MYCN, tissue-specific enhancers, and additional TFs constitutes a core regulatory circuitry that underlies maintenance of lineage-related NB gene expression programs.

In *MYCN*-amplified NB, the expression of *MYCN* itself is regulated by large superenhancers that map to the *MYCN* amplicon (13). This has spurred renewed efforts to target MYCN transcription through inhibition of transcriptional coregulators that are enriched at enhancers and superenhancers, including the BET-bromodomain transcriptional coactivators and some of the transcriptional cyclin-dependent kinases (CDK7/9/12/13) (14–18). In NB and other cancers, targeting these transcriptional components leads to selective downregulation of superenhancer-associated genes, such as *MYC* or *MYCN*, that are characterized by high transcription levels and rapid turnover of RNA. These observations have spurred further preclinical investigation of transcriptional inhibition in NB. However, as almost all cells have superenhancers at key identity genes, it remains unclear how transcriptional inhibition can achieve selectivity, how Myc addiction is subverted by transcriptional inhibition to kill tumor cells, and how a therapeutic strategy for transcriptional inhibition can be implemented.

Here, in order to accelerate the clinical implementation of transcriptional inhibitors in NB, we investigate the ability of CYC065 (fadraciclib), a selective inhibitor of CDK9 and CDK2 that has reached clinical early phase trials, to selectively target *MYCN-*amplified tumors via multiple mechanisms. CYC065 (19) and its analog CCT68127 (20) were discovered in a research program aimed at identifying derivatives of seliciclib (21) with greater potency, solubility, selectivity, and metabolic stability (22).

## Results

### CDK9 inhibition downregulates MYCN and is selective against MYCN-amplified NB.

**Figure 1:**
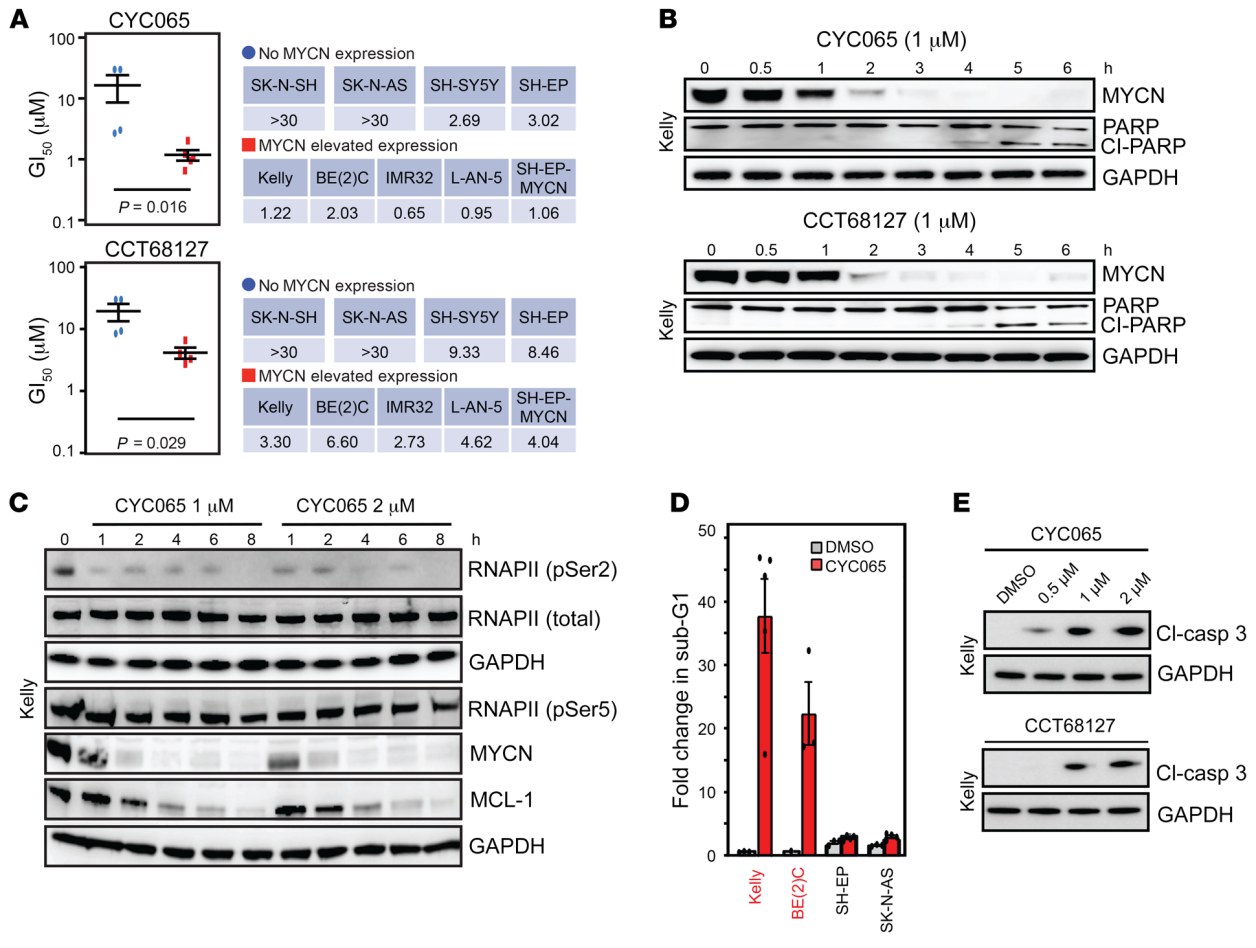
CDK9 and CDK2 are selectively essential for *MYCN*-amplified NB. (**A**) GI_50_ of CCT68127 and CYC065 in a panel of NB cells. Cells were treated for 8 hours, washed off, and replaced with normal growth medium. GI_50_ values (μM) were calculated after 72 hours (*n* = 3). Significance was calculated using 2-tailed, unpaired Student’s *t* test. (**B**) Kelly cells were treated with CYC065 or CCT68127 for 0.5 to 6 hours (1 μM). Immunoblots illustrate expression of PARP cleavage (*n* = 2). (**C**) Immunoblots showing expression of p-RNAPII-Ser2 and p-RNAPII-Ser5, MYCN, and MCL-1 at the indicated times after treatment with CYC065 (1–2 μM, 1–8 hours) in Kelly cells (*n* = 2). (**D**) Flow cytometry analysis showing sub-G1 level of *MYCN*-amplified (Kelly, BE(2)C) and nonamplified (SH-EP, SK-N-AS) cells in response to CYC065 (1 μM; 8 hours). Data are represented as mean ± SD of 3 independent experiments. (**E**) Kelly cells were treated with CYC065 or CCT68127 at the indicated concentrations (0.5–2 μM) for 6 hours. Immunoblots depict expression of cleaved caspase-3 (*n* = 2).

We evaluated a series of clinical candidate drugs and tool kit compounds with potent and selective activity against CDK9 and varying activity against other CDKs. We observed that compounds with prominent activity against CDK9 are efficient in downregulating MYCN to varying degrees and inducing apoptotic cell death, as indicated by induction of poly (ADP-ribose) polymerase (PARP) cleavage (Supplemental Figure 1A and Supplemental Figure 2B; supplemental material available online with this article; https://doi.org/10.1172/JCI134132DS1). Of these compounds, we selected the chemical probe CCT68127 (22) and its further optimized derivative CYC065 (19), which is in early phase clinical trials — both with significant selectivity for CDK9/2 (refs. 19, 20, 23 and Supplemental Figure 2, A and B). We evaluated CYC065 and CCT68127 across a set of NB cell lines (Figure 1A and Supplemental Figure 2, D and E) varying in *MYCN* amplification and protein levels and characterized for MYCN or MYC dependence (ref. 15 and Table 1). MYCN-driven cell lines exhibited time- and concentration-dependent growth inhibition, increased cell death (by sub-G1 population), and induction of apoptosis (caspase-3 and PARP cleavage), resulting in loss of cell viability and, with prolonged treatment, blockade of colony formation (Figure 1, B–E, and Supplemental Figure 2, C and E–H). These effects occurred at concentrations of CYC065 and CCT68127 coinciding with a reduction in MYCN protein and a reduction in phosphorylation of RNAPII serine 2 (RNAPII Ser2P), a canonical substrate of CDK9 (Figure 1C and Supplemental Figure 2H). Notably, in non–*MYCN*-amplified NB (SH-EP, SH-SY5Y, and SK-N-AS cells), CYC065 still potently reduced RNAPII Ser2P at compound concentrations that had no or little impact on apoptosis (Supplemental Figure 2, I and J), suggesting that transcriptional inhibition is not lethal in these non–*MYCN*-amplified cells.

At the cellular concentration at which cell growth is inhibited by 50% (GI_50_), CYC065 and CCT68127 primarily caused cell growth arrest and apoptosis in *MYCN*-amplified NB cells (Figure 1, A and B, and Supplemental Figure 2, D–F). Contrary to a report in lung cancer cells that CCT68127 caused anaphase catastrophe (18), NB cells treated with CYC065 at low concentrations exhibited intact mitotic spindle formation and, at higher concentrations or with prolonged treatment, exhibit DNA fragmentation consistent with apoptosis (Supplemental Figure 3A). These data suggested that, at concentrations close to the GI_50_ and therefore relevant to the mechanism of action for CDK9 and CDK2, CYC065 treatment results in growth arrest and apoptosis in *MYCN*-amplified cells.

### Cell death upon CDK9 inhibition is enhanced by concomitant blockade of CDK2 activity.

**Figure 2:**
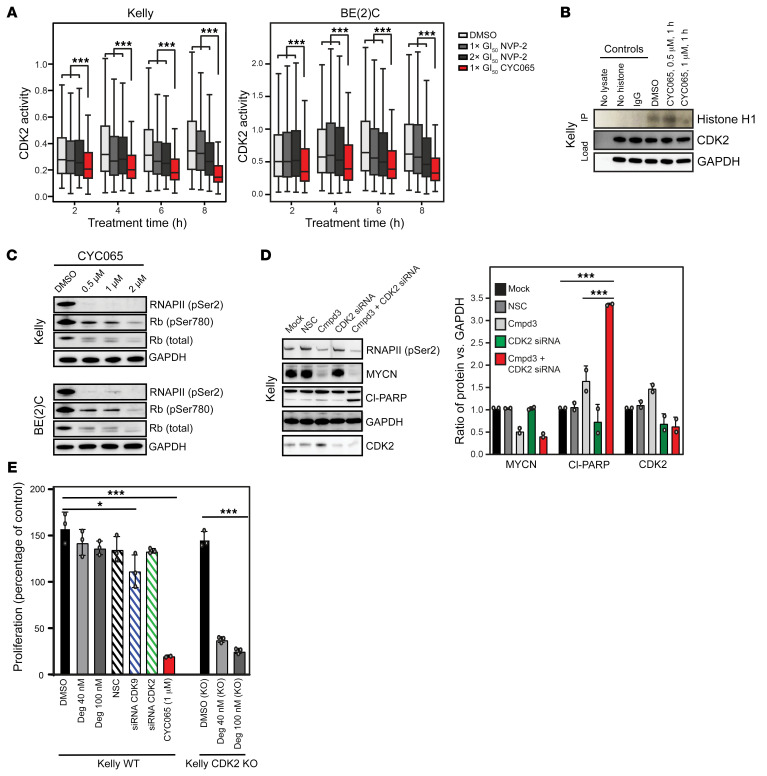
CDK9 and CDK2 synergistically maintain *MYCN*-amplified NB cells. (**A**) CDK2 activity is obtained by measuring the cytoplasmic-to-nuclear ratio of DHB-mVenus. Cell nuclei were identified using DAPI staining. Bold lines represent median, boxes represent the interquartile range (IQR), whiskers represent 1.5 times the interquartile range, and outliers are not shown. Welch’s 2-tailed *t* test with Benjamini and Hochberg correction for multiple comparison. ***P < 1 × 10^–8^. (**B**) Kelly cells were treated with CYC065 or DMSO and harvested after 1 hour. CDK2 complexes were immunoprecipitated from cell lysates followed by an in vitro kinase assay using histone H1 as a substrate (*n* = 2). (**C**) Kelly and BE(2)C cells were treated with CYC065 for the indicated concentrations (0.5–2 μM) for 8 hours. Immunoblots show expression of the Rb protein (*n* = 2). (**D**) Immunoblots and bar plots showing expression of MYCN and cleaved PARP when cells were treated with compound 3 (Cmpd 3) at 1 × GI_50_ and/or siRNA directed to CDK2. Data are represented as mean ± SD of 2 independent experiments. Two-tailed, unpaired Student’s *t* test with Benjamini and Hochberg correction for multiple comparisons. ***P < 0.001. (**E**) Proliferation of NB cells quantified using a CellTiter-Glo assay. Kelly cells with CRISPR Cas9–mediated knockout of CDK2 (KO) or endogenous (WT) CDK2 were treated with CYC065 (8 hours), Deg (THAL-SNS-032, 8 hours), and siRNA against CDK9 or CDK2 for 48 hours. Data are represented as mean ± SD of 3 independent experiments. Two-tailed, unpaired Student’s *t* test with Benjamini and Hochberg correction for multiple comparisons. *P < 0.05; ***P < 0.001.

We observed that, in comparison with monoselective inhibitors of CDK9, such as compound 3 (24), which is only able to induce PARP cleavage at high concentrations, CYC065 caused extensive apoptotic cell death at cellular GI_50_ concentrations in *MYCN*-amplified NB cells, presumably due to concomitant inhibition of CDK2, itself a major regulator of apoptotic cell death. Apoptosis occurred concomitant with a marked reduction in MCL-1 (Supplemental Figure 1A and Supplemental Figure 2H), a transcriptional target of CDK9 with prominent prosurvival activity dependent on CDK2 phosphorylation (25). Using a fluorescence-based cellular sensor that measures phosphorylation of substrate by CDK2 (26), we confirmed that, at the GI_50_ concentration, CYC065 reduced CDK2-driven kinase activity (Figure 2A), blocked phosphorylation of histone H1 (a direct substrate of CDK2, Figure 2B), and upregulated the proapoptotic CDK2 targets (Supplemental Figure 4A). In contrast, NVP-2, a highly potent and selective CDK9-only inhibitor (27), failed to decrease CDK2 substrate phosphorylation to the same degree (Figure 2A).

With extended exposure to CYC065 (8 hours), we also observed a reduction in phosphorylation of Rb serine 780 (Figure 2C) and an accumulation of total and phosphorylated p53 (Supplemental Figure 2H, Supplemental Figure 4, B–D, and Supplemental Figure 5H), both known consequences of CDK2 inhibition (15). In CDK9-inhibited and MYCN-dependent cells, activation of apoptotic cell death is CDK2 dependent, as the monoselective CDK9 chemical probe (compound 3) (24), the clinical monoselective CDK9 inhibitor BAY1143572 (28) (atuveciclib), and knockdown of CDK2 with siRNA each failed to induce robust apoptosis (Figure 2D and Supplemental Figure 4, E and G). In contrast to monospecific inhibition of CDK9 or CDK2, the combination of selective CDK9 inhibition (Compound 3 or BAY1143572) with siRNAs directed at CDK2 resulted in enhanced PARP cleavage (Figure 2D and Supplemental Figure 4, E and G). Since the siRNA knockdown of CDK2 was modest, we performed CRISPR Cas9–mediated knockout of CDK2 in *MYCN*-amplified Kelly cells (Supplemental Figure 5G), which elicited minimal effects on apoptosis or cell cycle distribution (Supplemental Figure 4G), and in parental *MYCN*-amplified Kelly cells, selective chemical inhibition or genetic knockdown of CDK9 or CDK2 alone failed to phenocopy the growth inhibitory effects of CYC065 (Figure 2E and Supplemental Figure 4G). Finally, we observed that only in CDK2-knockout cells did compound 3 treatment or CDK9 degradation using THAL-SNS-032 (27) (a potent and selective CDK9 degrading PROTAC) result in an increase in sub-G1 apoptotic cells (Supplemental Figure 4, F and G) and growth inhibitory effects (Figure 2E). Taken together, these data confirm that in the setting of CDK9-induced MYCN blockade, activation of apoptotic cell death in *MYCN*-amplified NB requires concomitant diminution of CDK2 activity.

### CDK9 inhibition blocks nascent transcription of MYCN and other highly transcribed genes.

**Figure 3:**
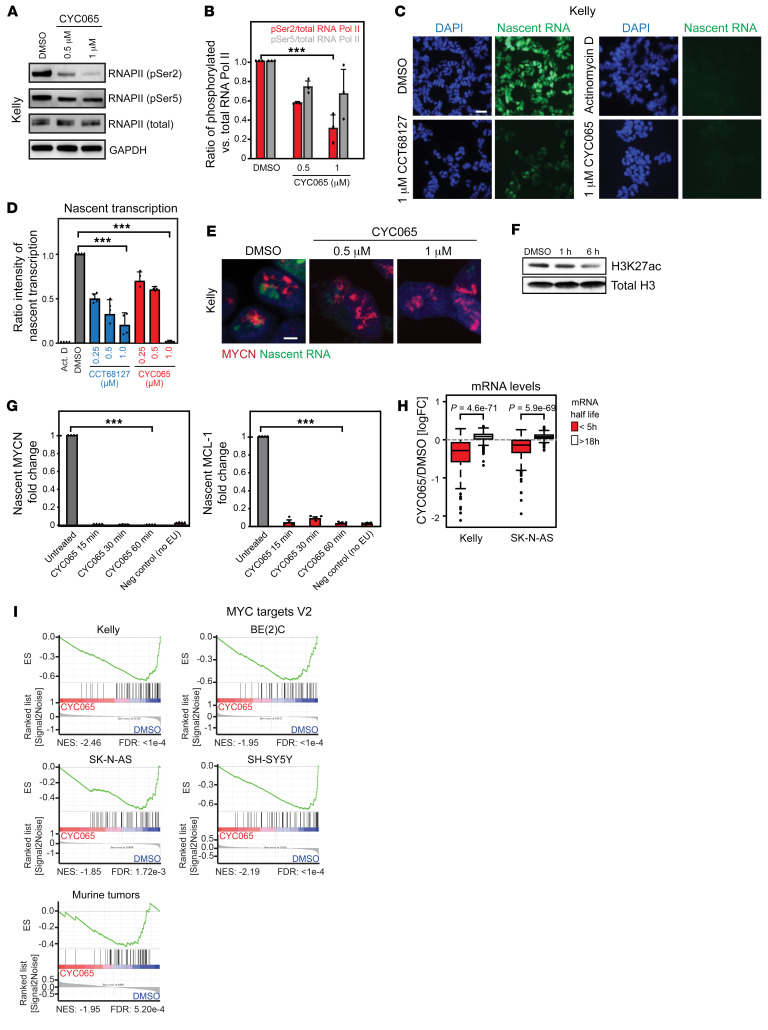
Inhibition of CDK9 blocks transcription of *MYCN* and genes with short half-lives. (**A** and **B**) Immunoblot and graph showing effects of treatment with CYC065 (6 hours) on phosphorylation of RNAPII at Ser2 and Ser5 at the indicated concentrations in Kelly cells. Data are represented as mean ± SD of 3 independent experiments. Two-tailed, unpaired Student’s *t* test with Benjamini and Hochberg correction for multiple comparisons. ***P < 0.001. (**C** and **D**) Click-IT assay showing effect of CYC065 or CCT68127 (0.25–1 μM, 1 hours) on the abundance of newly synthesized nascent RNA in Kelly cells as illustrated in green fluorescence (**C**) and graph. Scale bar: 10 μm. (**D**). Data are represented as mean ± SD of 4 independent experiments. Two-tailed, unpaired Student’s *t* test with Benjamini and Hochberg correction for multiple comparisons ***P < 0.001. Scale bar: 10 μm. (**E**) Immunofluorescence showing newly synthesized nascent RNA (green) as described in **C** and FISH of MYCN gene– (red) and DAPI-stained nucleus (blue) following 1 hour treatment with CYC065 in Kelly cells (*n* = 3). (**F**) Immunoblot showing level of H3K27ac after treatment with CYC065 (1 μM) for 1 hour and 6 hours (*n* = 1). (**G**) 1PCR analyses showing levels of MYCN and MCL-1 genes extracted from fluorescently labeled nascent RNA in Figure 3C. Data are represented as mean ± SD of 4 independent experiments. Two-tailed, unpaired Student’s *t* test with Benjamini and Hochberg correction for multiple comparisons. ***P < 0.001. (**H**) Box plot documenting gene expression changes after CYC065 treatment (1 μM, 1 hour) of genes with short (<5 hours, *n* = 386) and long (>18 hours, *n* = 380) mRNA half-lives (58). Two-tailed, unpaired Wilcoxon’s rank sum test. (**I**) GSEA in *MYCN*-amplified (Kelly, BE(2)C), non–*MYCN*-amplified (SK-N-AS, SH-SY5Y) NB cell lines and tumors from TH-*MYCN* mice after treatment with CYC065. Shown is the MYC target gene V2 gene set from the Hallmark collection of the MSigDB.

Together with cyclin T1, CDK9 forms P-TEFb, which promotes transcriptional elongation via direct phosphorylation of Ser2 in the carboxy-terminal repeat (CTD) of RNAPII (29–33). Consistent with its property of inhibiting CDK9, CYC065 at a GI_50_ concentration blocked phosphorylation of RNAPII Ser2, while RNAPII Ser5, a target of CDK7, was inhibited only at higher concentrations (Supplemental Figure 1A and Figure 3, A and B). Short-term treatment (1 hour) with CYC065 or CCT68127 globally reduced, but did not totally block, nascent RNA synthesis, as determined by in situ staining (Figure 3, C and D). In comparison, actinomycin D (ActD), which inhibits transcription initiation, completely abrogated nascent transcription at a 0.5 μg/mL concentration. These results suggest that CDK9 inhibition at least partially inhibits global transcription elongation.

Using high-resolution imaging, we noticed consistent overlap of nascent transcription foci at the *MYCN* amplicon (visualized by DNA FISH) that were abrogated by the exposure to the GI_50_ concentration of CYC065 (Figure 3E). Loss of *MYCN* transcript temporally coincided with global loss of nascent transcription and chromatin acetylation, as evidenced by H3K27ac levels (Figure 3F). This is consistent with the overall role of Myc proteins in amplifying gene expression (8, 9) and maintaining open chromatin (34). Indeed, nascent transcription of highly expressed, high-turnover transcripts such as *MYCN* and *MCL-1* was almost completely inhibited within 15 minutes of CYC065 treatment (Figure 3G), and overall, short half-life genes such as *MYCN* (Supplemental Figure 5A) were selectively depleted from the cellular mRNA pool (Figure 3H). In contrast, CYC065 had only modest effects on MYCN protein turnover (Supplemental Figure 5, B and C). MYCN loss was phenocopied by selective degradation of CDK9 by THAL-SNS-032 and genetic depletion of CDK9 or to a lesser extent CDK7 (Supplemental Figure 5, D–F), consistent with a general sensitivity of *MYCN* to transcriptional inhibition. Knockdown of CDK2 did not affect MYCN levels (Figure 2D, Supplemental Figure 4F, and Supplemental Figure 5G). Notably, when MYCN was exogenously expressed, its levels were no longer sensitive to CYC065 or CCT68127 (Supplemental Figure 5H). These data confirm that nascent transcription of the *MYCN* amplicon is uniquely sensitive to transcriptional perturbation and to inhibition of CDK9.

Further analysis of mRNA levels upon CYC065 treatment across a panel of *MYCN-*amplified NB cell lines as well as tumors from the MYCN-driven TH-*MYCN* mouse model revealed a selective depletion of Myc target gene expression (Figure 3I and Supplemental Figure 6E). This effect was confirmed at individual genes, on a Myc target luciferase reporter, and by showing depletion of MYCN from a target gene promoter by ChIP (Supplemental Figure 5, A, I, and J). Although MYCN depletion was much more pronounced in *MYCN*-amplified NB (Supplemental Figure 6B), depletion of Myc-driven housekeeping gene expression was also consistently observed in non–*MYCN-*amplified NB (Figure 3I and Supplemental Figure 6, A–E), suggesting that irrespective of *MYCN* amplification status, CDK9 inhibition targets canonical Myc target gene signatures associated with growth and biogenesis.

### MYCN enhancer invasion shapes NB-specific responses to CYC065.

Although CYC065 downregulated canonical Myc target gene expression in both *MYCN* and non–*MYCN-*amplified NB, its highly selective effects on *MYCN*-amplified NB growth spurred us to further investigate why and how MYCN expression or amplification induces this dependency in NB. We considered 2 hypotheses. First, in *MYCN*-amplified NB, coamplification of the *MYCN* gene locus and of distal regulatory regions is frequently observed within a roughly 1 Mb amplicon (13, 16). Second, when amplified, hyperabundant MYCN protein saturates high-affinity binding sites at promoters of housekeeping genes and in turn invades lower affinity sites at the promoters and enhancers of tissue-specific genes (13).

**Figure 4:**
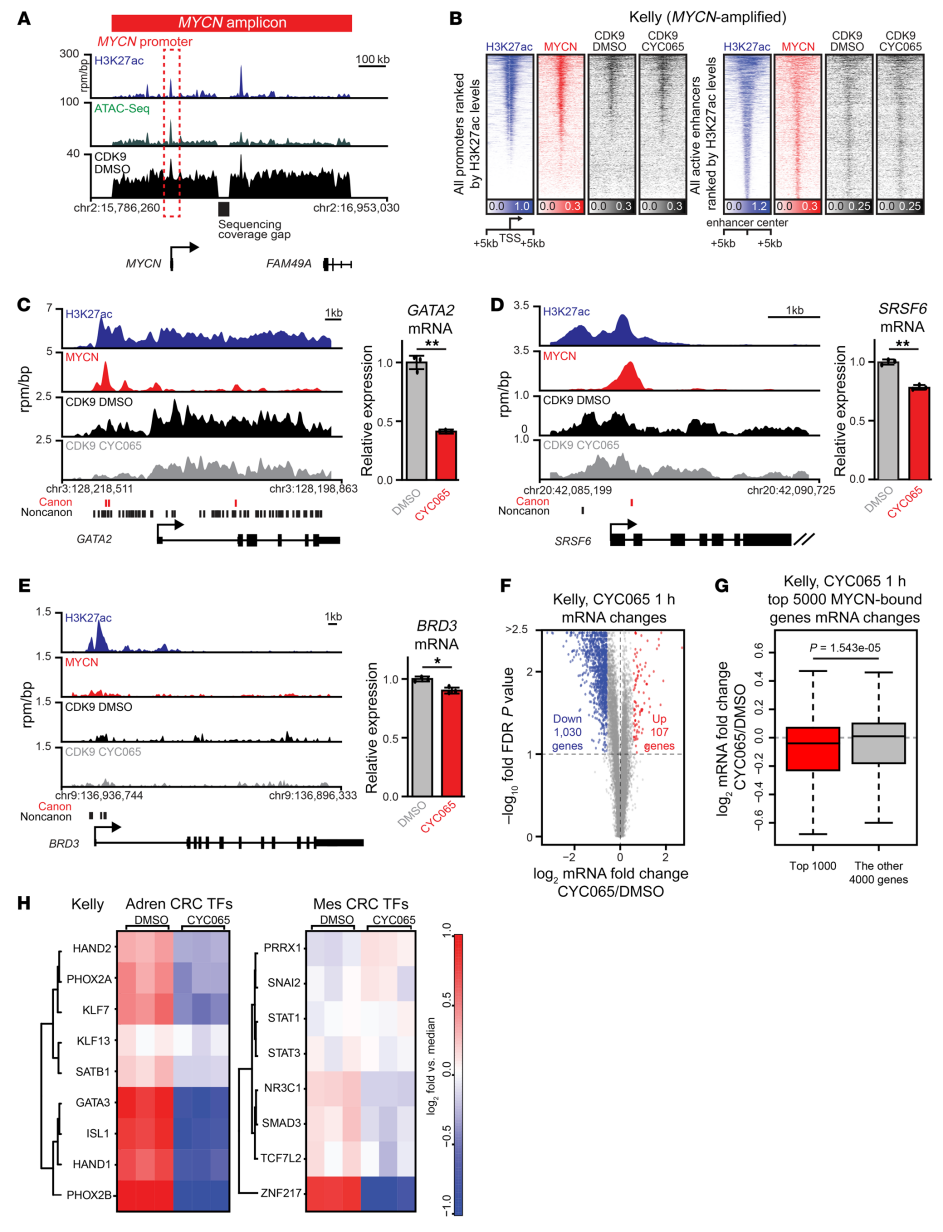
Pharmacologic blockade of CDK9 targets MYCN-dependent transcriptional landscape. (**A**) Gene tracks of chromatin accessibility (shown by ATAC-Seq, green), active chromatin marker: H3K27ac (blue) and CDK9 (black) occupancy at *MYCN* amplicon in Kelly cells. (**B**) Heatmaps of H3K27ac (blue), MYCN (red), and CDK9 (black) occupancy at all promoters (left) or enhancers (right) ranked by H3K27ac signal. Each row of heatmaps suggests 1 promoter region or enhancer region. The middle of heatmaps indicates the TSS or enhancer centers. (**C**–**E**) Left: gene tracks of H3K27ac (blue), MYCN (red), and CDK9 (black) (±CYC065) occupancy at individual loci. ChIP-Seq occupancy is provided in units of rpm/bp. Canonical MYCN-binding sites (red lines) and noncanonical MYCN-binding sites (black lines) are indicated below gene tracks. Right: bar plots of corresponding gene expression normalized to control showing effect of CYC065 (1 μM; 1 hour) treatment. Data are represented as mean ± SD. Two-tailed Student’s *t* test. *P < 0.05; **P < 0.01. (**F**) Scatter plot of log_2_ gene expression (FPKM) fold changes (CYC065; 1 μM; 1 hour) treatment vs. DMSO control (*x* axis) versus significance of the change (*y* axis, –log_10_ FDR value). Genes with 1.5-fold or greater change in expression at an FDR of 0.1 or less are considered differentially expressed (blue and red). (**G**) The top 5000 transcriptionally active, expressed, and MYCN-associated genes are ranked by MYCN load (promoter + enhancer MYCN). Box plot implicating the log_2_ mRNA fold change of the top 1000 genes and the log_2_ mRNA fold change of the other 4000 genes. Two-tailed Student’s *t* test. (**H**) Heatmap indicating the mRNA log_2_ FPKM fold change from the FPKM median of TFs in adrenergic (Adren) and mesenchymal (Mes) core regulatory circuitries, with CYC065 (1 μM; 1 hour) treatment in Kelly cells.

To test these 2 hypotheses, we performed ChIP-Seq for CDK9 and integrated its genome-wide occupancy with our MYCN and chromatin landscapes (13) in *MYCN*-amplified NB. Addressing the first hypothesis, we identified strong enrichment for CDK9 at both the *MYCN* promoter and the distal superenhancer (Figure 4A). Investigating the second hypothesis of MYCN global effects, we observed widespread binding of MYCN to both promoters and enhancers, coincident with binding of CDK9 (Figure 4B). We and others have shown that the effect of Myc protein transcriptional regulation at target genes is proportional to the amount of Myc present at the promoter and nearby enhancers (13, 35). At individual loci in Kelly cells, we observed a concentration-dependent relationship between overall MYCN occupancy and the magnitude of expression downregulation caused by CYC065 treatment at 1 hour. *GATA2*, a developmental TF associated with the adrenergic state of MYCN-driven NB (10, 12), possesses abundant MYCN and CDK9 binding at upstream enhancers. Its gene expression was potently downregulated by CYC065 without substantial perturbation of CDK9 occupancy (Figure 4C). In contrast, *SRSF6* and *BRD3*, genes with decreasing MYCN and CDK9 promoter/enhancer occupancy, respectively, exhibited more modest sensitivity to CYC065 (Figure 4, D and E). Overall, CYC065 treatment leads to global downregulation of gene expression, as significantly downregulated genes outnumber upregulated genes by approximately 10:1 (Figure 4F). Ranking the top 5000 genes by MYCN occupancy, we found that CYC065’s effect on gene expression was concordant with MYCN occupancy (Figure 4G). Consistent with our 2 hypotheses, these data suggest that CDK9 occupies own superenhancer and that CYC065 treatment selectively downregulates genes with elevated MYCN binding at their promoters and enhancers.

Previously, NB tumors have been shown to adopt and interconvert between 2 lineage-derived and transcriptionally encoded states (adrenergic or mesenchymal CRCs), expression of which is maintained by interactions between groups of TFs and enhancers and superenhancers (10–12). Interestingly, TFs that make up the adrenergic CRC show strong interactions with MYCN. MYCN binds the enhancers of these TFs and cobinds with these TFs at other enhancers across the genome, and knockdown of these adrenergic CRC TFs downregulates MYCN regulation of tissue-specific gene expression (10–12). With CYC065, we observed a selective depletion of CRC TFs driving the adrenergic state of NB, as compared with the perturbation of mesenchymal master regulator TFs (Figure 4H). Overall, these data are consistent with the ability of CYC065 to selectively deplete MYCN and thus preferentially downregulate these highly MYCN-occupied genes that crosscorrelate with the MYCN-associated adrenergic gene expression program that is essential for NB growth.

### CYC065 targets the adrenergic state.

**Figure 5:**
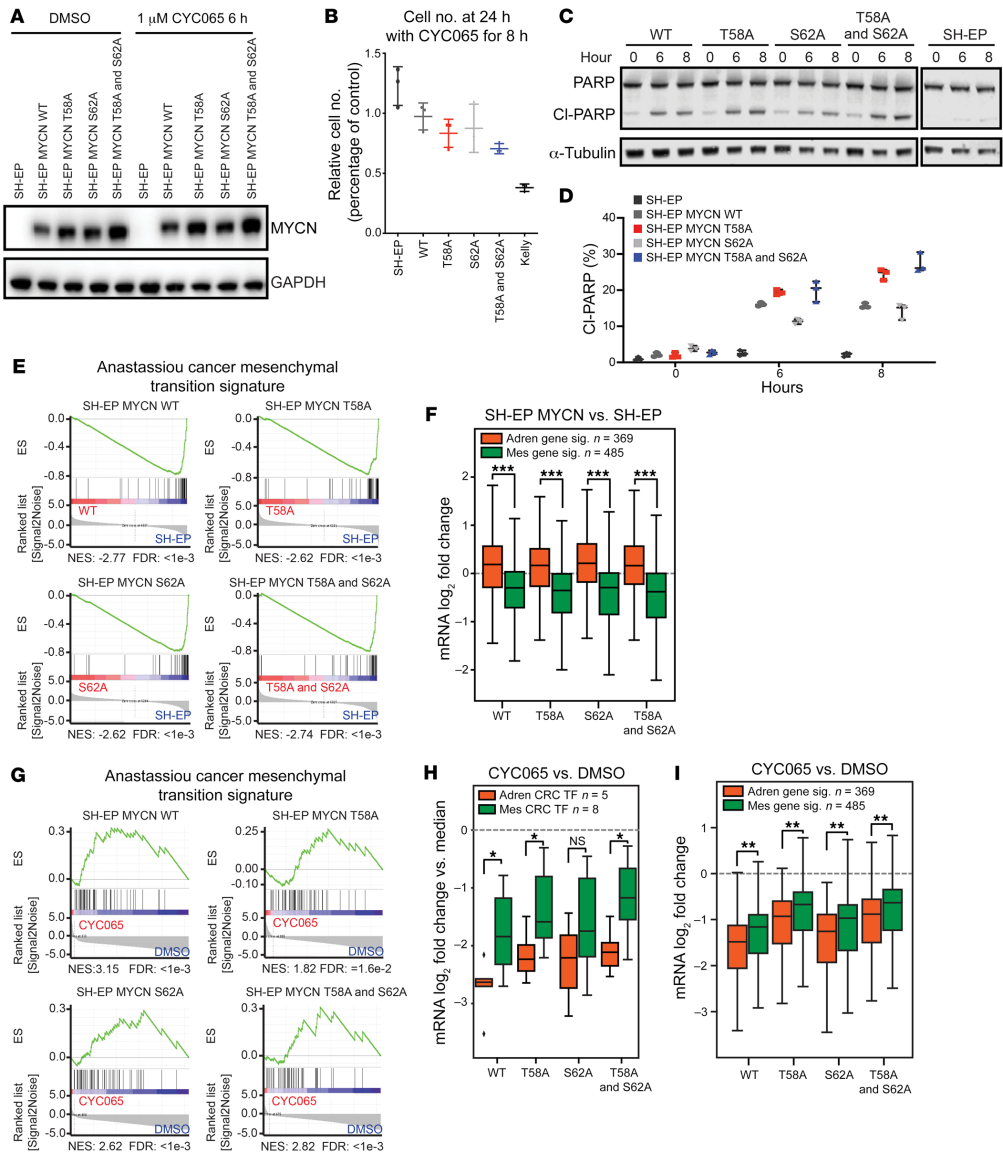
CYC065 directly blocks MYCN-driven adrenergic cell identity. (**A**) Immunoblots indicate stable MYCN expression in SH-EP MYCN system with CYC065 treatment (1 μM; 6 hours). (**B**) Potency against SH-EP and SH-EP MYCN cells in vitro. Cells are treated with 1 μM CYC065 for 8 hours followed by two PBS washes. Relative cell counts were calculated using CellTiter-Glo assays. Data are represented as mean ± SD of 3 independent experiments. (**C**) Immunoblots depict effect of 1 μM CYC065 treatment in SH-EP and SH-EP MYCN cells for 6 hours and 8 hours. (**D**) Dot plot showing quantification of PARP and cleaved PARP (Cl-PARP) in **C**. Data are represented as mean ± SD of 3 independent experiments. (**E**) GSEA in SH-EP and SH-EP MYCN cell lines. Anastassiou cancer mesenchymal transition signature is from the Hallmark collection of the Molecular Signatures Database. (**F**) Box plot showing SH-EP MYCN mRNA log_2_ fold change of adrenergic (ADRN) genes and mesenchymal (MES) genes compared with SH-EP cells. Bold lines represent median, boxes represent interquartile range, and whiskers represent 1.5 times the interquartile range. Outliers are not shown. Welch’s 2-tailed *t* test, Benjamini and Hochberg correction for multiple comparisons. ***P < 1 × 10^–8^. (**G**) GSEA in SH-EP and SH-EP MYCN cell lines after treatment with CYC065 (1 μM; 6 hours). (**H**) Box plot showing mRNA log_2_ fold change of TFs from median in adrenergic and mesenchymal core regulatory circuitries in SH-EP MYCN cells, which is caused by CYC065 (1 μM; 6 hours) treatments. Outliers are represented as dots. Welch’s 2-tailed *t* test and Benjamini and Hochberg correction for multiple comparisons. *P < 0.05. (**I**) Box plot showing CYC065 (1 μM; 6 hours) treatment and SH-EP MYCN mRNA log_2_ fold change of adrenergic genes and mesenchymal genes. Outliers are not shown. Welch’s 2-tailed *t* test, Benjamini and Hochberg correction for multiple comparisons. **P < 1 × 10^–4^.

Observing that CYC065-mediated downregulation of MYCN selectively targets TFs defining the adrenergic NB state, we next sought to see whether the converse were true — would MYCN overexpression convert mesenchymal NB into a more adrenergic state? Here, we used the SH-EP NB cell line, which has demonstrated mesenchymal identity (12) and no evidence of *MYCN* genomic amplification or expression. Using retroviral transgene expression systems, we created stable SH-EP cells overexpressing WT MYCN as well as phosphorylation-deficient mutants (T58A, S62A, and the combined T58A S62A double mutant), all under the control of an exogenous promoter (Figure 5A). Phosphorylation of Myc proteins at both the highly conserved T58 and S62 residues is required for their proteasome-dependent turnover, and these mutants (especially the T58A) are considered to be more stable and oncogenic (36). In contrast to endogenously *MYCN*-amplified NB, treatment with CYC065 failed to decrease exogenous MYCN levels in these cells (Figure 5A). Across MYCN phosphorylation-deficient mutant variants, MYCN binding at promoters and enhancers was unchanged upon CYC065 treatment (Supplemental Figure 7, A and B), with the exception of the T58A S62A double mutant, which exhibited a global decrease in MYCN occupancy (Supplemental Figure 7, C and D). These data are consistent with our prior conclusions that CYC065 selectively targets nascent *MYCN* transcription specifically in the context of endogenous *MYCN* amplification.

Overexpression of MYCN in SH-EP cells increases cellular growth rate, but also renders these cells more sensitive to growth inhibition induced by CYC065 treatment (Figure 5B, Supplemental Figure 2D, and Supplemental Figure 7E). This result is surprising, given that MYCN levels were not depleted in the context of exogenous MYCN expression. The effect was more obvious in the hyperstabilized MYCN phosphorylation-deficient mutants. For mutants containing T58A, sensitivity to CYC065 treatment correlated with increased PARP cleavage (Figure 5, B–D). These observations led us to hypothesize that MYCN overexpression altered the underlying cell state of SH-EP cells, potentially inducing a mesenchymal to adrenergic cell state transition. Using RNA-Seq, we profiled the transcriptomes of the various MYCN-overexpressing SH-EP cells and compared them with parental SH-EP cell gene expression profiles. Across all MYCN overexpression variants, we observed downregulation of genes encoding for mesenchymal identity as defined from more general molecular signature databases (Figure 5E) and specifically defined in mesenchymal NB subtypes (Figure 5F). Loss of mesenchymal gene expression coincided with an increase in expression of adrenergic-specific NB genes (Figure 5F). Using cell count–normalized gene expression, we again observed that CYC065 treatment globally downregulated gene expression, with more than 95% of active genes downregulated. Only a small number of lowly expressed genes (<5 FPKM) are appreciably upregulated (Figure 5G). Among downregulated genes, mesenchymal gene signatures were the least downregulated (Figure 5G), suggesting that mesenchymal-encoding genes are not strongly occupied by MYCN. This finding is reinforced by the data in Figure 5, H and I, showing that adrenergic signatures are more strongly downregulated than mesenchymal signatures by CYC065. These data suggest that MYCN overexpression converts NB to an adrenergic state and that CYC065 is able to target this state independently of any direct action against MYCN by selectively downregulating MYCN-induced adrenergic gene expression.

### CYC065 selectively inhibits growth of MYCN-amplified NB in vivo.

**Figure 6:**
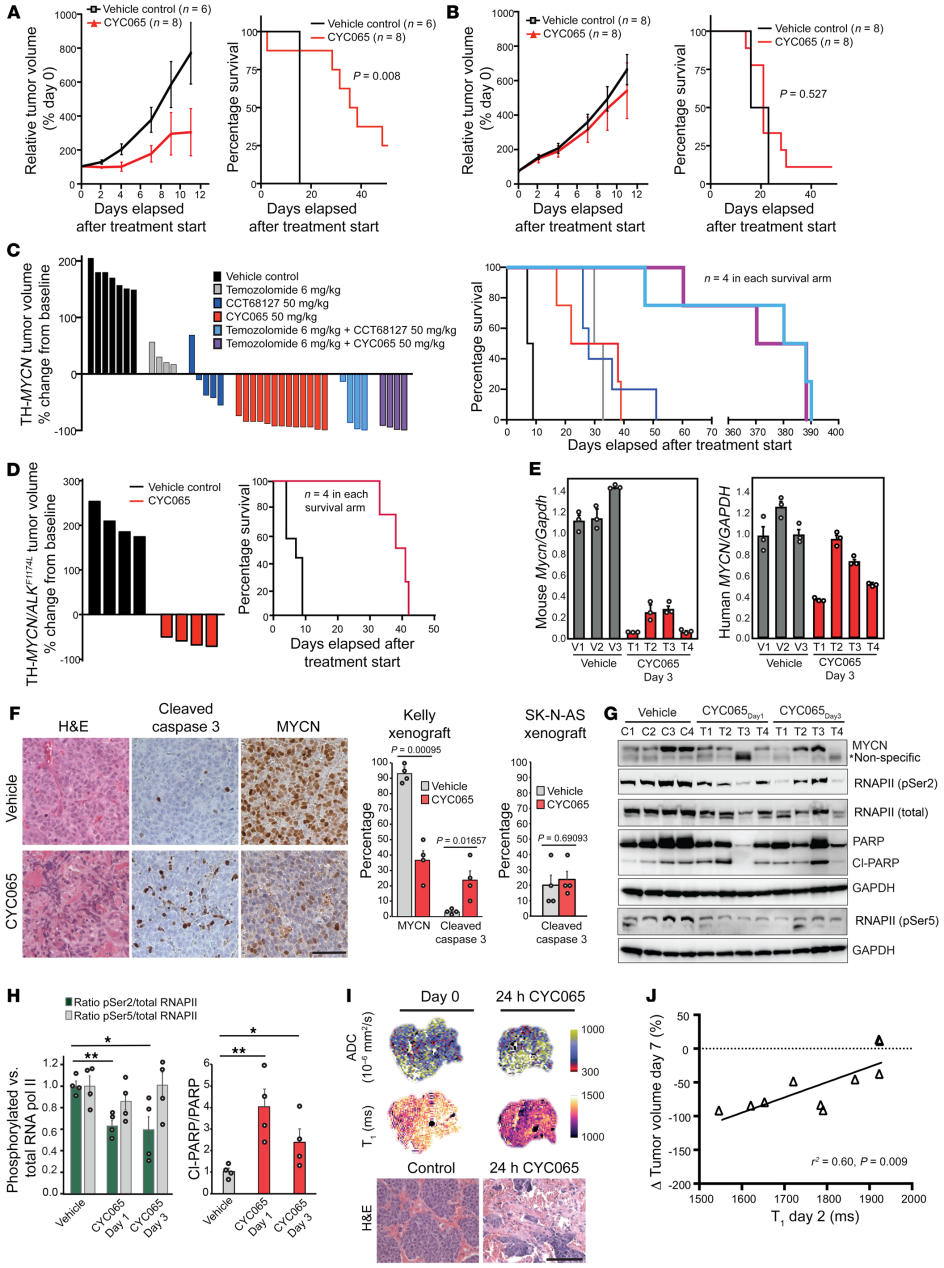
CYC065 and CCT68127 inhibit MYCN-driven NB in vivo. Effects of CYC065 on the growth and survival of Kelly (*MYCN* amplified) (**A**) and SK-N-AS (nonamplified) (**B**) NB xenografts in mice. Data are expressed as mean ± SEM (log-rank Mantel-Cox test with a 5% level of significance). (**C**) Waterfall plot documenting relative changes in tumor volume at day 7 in the TH-*MYCN* GEM model. All treatment arms versus control: *P* < 0.001, 2-tailed, unpaired Student’s *t* test incorporating Bonferroni’s correction (*n* = 5) with a 1% level of significance. Kaplan-Meier plot documenting survival of TH-*MYCN* mice. All treatment arms versus control: *P* < 0.01; and CYC065 or CCT68127 alone versus combination with temozolomide: *P* = 0.02 (log-rank Mantel-Cox test with 5% level of significance. (**D**) Waterfall plot documenting relative changes in tumor volume at day 7 in the TH-*ALK^F1174L^/MYCN* GEM model: *P* < 0.001, 2-tailed unpaired Student’s *t* test with 5% level of significance. Kaplan-Meier plot documenting survival of TH-*ALK^F1174L^/MYCN* mice: *P* < 0.01, log-rank Mantel-Cox test with a 5% level of significance) (**E**) Quantitative RT-PCR analyses showing levels of murine and human *MYCN* RNA in the TH-*ALK^F1174L^/MYCN* tumor following treatment with CYC065 for 3 days (*n* = 3). (**F**) Representative images and quantitative analysis of H&E and immunohistochemical staining for cleaved capsase-3 and MYCN in the harvested tumors from **A** and **B**. Scale bar: 50 μm. (**G** and **H**) Immunoblot analyses of individual tumors from TH-*MYCN* model treated with CYC065 for 1 or 3 days. Data are represented as mean ± SD of 4 independent experiments. Two-tailed unpaired Student’s *t* test with Benjamini and Hochberg correction for multiple comparisons. *P < 0.05; **P < 0.01. (**I**) Parametric functional MRI maps showing reduction of tumor spin lattice relaxation time T_1_ and an increase in ADC 24 hours after treatment with 50 mg/kg CYC065, and their corresponding H&E staining. Scale bar: 100 μm. (**J**) Correlation between native tumor T_1_ measured 24 hours after treatment with 50 mg/kg CYC065 or CCT68127 (percentage of pretreatment value) and relative changes in tumor volume following treatment with 50 mg/kg CYC065 or CCT68127.

As CYC065 is currently in early phase clinical evaluation in adults, we investigated its efficacy in murine models of NB. CYC065 induced significant tumor growth inhibition and increased overall survival in mice carrying *MYCN*-amplified Kelly NB tumor xenografts, but had weaker effects against non–MYCN-expressing SK-N-AS NB tumor xenografts (Figure 6, A and B), consistent with the modest effect on c-MYC levels (Supplemental Figure 8F). CYC065 had no effect on H128 tumor xenograft (Supplemental Figure 8A), which is a non–Myc-driven small cell lung cancer (9). In the extensively studied TH-*MYCN* murine model of NB in which MYCN is expressed under control of the tyrosine hydroxylase promoter, we administered CYC065 either orally or by intraperitoneal injection (Figure 6C and Supplemental Figure 8, B and C). Here, single-agent CYC065 treatment resulted in robust inhibition of tumor growth, and together with the DNA-damaging agent temozolomide, which is commonly used in the setting of treatment-refractory NB, we observed tumor eradication and remarkable extension of overall survival (Figure 6C). Finally, we tested CYC065 in an established transgenic model of NB, in which coexpression of hyperactivated anaplastic lymphoma kinase (ALK^F1174L^, a clinical mutation that cosegregates with *MYCN* amplification in NB patients) drives transcriptional activation of MYCN and formation of aggressive NB (37). In the TH-*ALK^F1174L^/*TH*-MYCN* genetically engineered mouse model (which expresses very high levels of murine Mycn as a consequence of direct activity of ALK on the endogenous *Mycn* promoter) (37), we observed tumor regression and a dramatic increase in overall survival compared with that seen with vehicle control (Figure 6D). These effects occurred at well-tolerated doses of CYC065 (Supplemental Figure 8D), suggesting a clear therapeutic index for CYC065 in the most highly aggressive MYCN-deregulated forms of NB.

We next determined whether CYC065 inhibition depleted endogenous MYCN, decreased transcriptional elongation, and induced apoptosis in our animal models. In *MYCN*-amplified Kelly NB tumor xenografts, we observed rapid loss of MYCN protein, induction of apoptosis, and decreased RNAPII Ser2P (Figure 6F and Supplemental Figure 8E). In TH-*ALK^F1174L^/TH-MYCN* tumors, we observed selective loss of the endogenous murine *Mycn* allele and a less pronounced effect on the exogenous human *MYCN* allele (Figure 6E). These data are consistent with our prior results (13) establishing CDK9 as a critical regulator of endogenous *MYCN* transcription. As with the previous in vitro studies, we observed increases in caspase-3 and PARP cleavage concomitant with MYCN loss in both Kelly NB tumor xenografts and TH-*MYCN* tumors following treatment with CYC065 (Figure 6, F–H). Pharmacodynamic effects of CYC065 treatment were also characterized by a change in the noninvasive functional MRI spin lattice relaxation time (T_1_) and apparent diffusion coefficient (ADC), which reflect a change in tissue integrity (38) and were indicative of further rapid reduction in tumor burden (Figure 6, I and J, and Supplemental Figure 8, G–I). Taken together, the data establish that the in vivo activity of CYC065 against MYCN-dependent NB tumor progression proceeds largely through transcriptional depletion of MYCN, leading to increased apoptosis and rapid loss of tumor burden. In other cancer models with Myc deregulated and nonderegulated subtypes, we observed similar trends, with selective inhibition of Myc-deregulated tumors coinciding with loss of Myc (Supplemental Figure 8J).

## Discussion

In this study, we establish that *MYCN*-amplified or *MYCN*-deregulated NB can be selectively targeted via combined CDK9/2 inhibition using CYC065, an orally bioavailable and clinically well-tolerated compound for which testing in the pediatric patient population is now warranted. In the preclinical setting, several multi-CDK inhibitors that also inhibit both have been shown to have varying ability to downregulate MYCN and kill NB cells (17, 39). Our data build upon previous preclinical (17, 39) and clinical studies of CDK inhibition in NB including studies of (a) dinaciclib, a broad spectrum, but poorly tolerated clinical inhibitor of CDKs (including CDK 1,2,5,9), which exhibited antiproliferative activity as a single agent and together with chemotherapy in NB cell lines and in vivo models; and (b) seliciclib (CYC202, *R*-roscovitine), an inhibitor of CDK2/5/7/9 that exhibited only partial activity against MYCN and was further limited by lack of potency and rapid clearance (19–23). Here, we show that the developmental clinical drug CYC065 — a potent and selective CDK9/2 inhibitor with enhanced pharmacokinetic and pharmacodynamics properties — is highly effective against NB. Furthermore, we demonstrate mechanistically that CYC065’s effects against high-risk MYCN-driven NB are a result of CDK9 inhibition resulting in selective loss of MYCN nascent transcription, which in turn leads to cell growth arrest and, in addition, sensitizes NB cells to apoptosis upon concomitant inhibition of CDK2 by the drug.

Recent work to characterize chromatin and transcriptional states in NB has more clearly defined how amplified *MYCN* invades enhancers and superenhancers of tissue-specific TFs to reshape gene expression and thereby enforce expression of a lineage-associated adrenergic state (10, 12). This invasion occurs only at oncogenic levels of MYCN and results in a highly interconnected and autoregulatory transcriptional circuitry in which MYCN regulates multiple adrenergic identity TFs (such as *GATA2*) that in turn also regulate both *MYCN* itself and tissue-specific enhancers invaded by MYCN (10, 11). Our data support a model in which CYC065 selectivity arises in part from the ability of CDK9 inhibition to collapse this transcriptional regulatory circuitry and break the autoregulatory feedback loop maintaining *MYCN* expression and adrenergic gene expression. Our results provide a mechanistic basis for the observed “transcriptional addiction” of these NB cells and further reinforce the emerging idea that drugs targeting core components of the transcriptional machinery can have a therapeutic index, especially in Myc-deregulated tumors (40). In addition to canonical enhancer or E-box–driven MYCN transcription, increased expression of MYCN could also be mediated by induction of *MYCNOS* (also known as *NYCM*), a regulatory antisense RNA, or other well-characterized lncRNAs located within the MYCN amplicon. *MYCNOS* transcript modulates the *MYCN* locus by recruiting chromatin modifiers and TFs, resulting in enhanced *MYCN* expression, and therefore logically could be inhibited by CYC065 treatment (41). Detailed study of these mechanisms is a future priority. Oncogenic dysregulation of Myc has also been directly associated with increased translational activity either through direct upregulation of rRNA and tRNA transcription (42–44), increased expression of core ribosomal proteins (45), or with perturbation of more selective mechanisms, such as targeting of eIF4A-mediated translational initiation (46). Additionally, rate-limiting control of translation taking place under conditions of normal tissue homeostasis is derepressed by oncogenic levels of Myc.

Additionally, the ability of NB tumors to interconvert between adrenergic and mesenchymal identity also implicates cell-state change as an anticipatable mechanism for achieving CYC065 resistance that could potentially be overcome by selective targeting of mesenchymal identity. Mesenchymal NB tumors are characterized by activated NOTCH signaling, and NOTCH-inhibiting γ secretase inhibitors have demonstrated some efficacy against NB models (47, 48). Whether combined targeting of adrenergic and mesenchymal identity is sufficient to establish antagonistic pleiotropy and further collapse NB tumors remains to be seen. Moreover, these data suggest that transcriptional inhibitors such as CYC065 will be more effective when used in combination rather than as a single agent — a conclusion supported by multiple observations of epigenetic and cell-state–mediated resistance to the BET-bromodomain family of transcriptional inhibitors (49). In NB, the strong combined effect we observed with CYC065 in combination with temozolomide, which is used for therapy-resistant NB, supports the addition of CYC065 as a means for selectively targeting MYCN-driven adrenergic identity.

Overall, we demonstrate that dual inhibition of CDK9 and CDK2 attacks MYCN dependence in NB through several mechanisms, including (a) selective blockade of CDK9 and superenhancer-regulated nascent endogenous *MYCN* transcription; (b) induction of CDK9/2-mediated proapoptotic pathways; and (c) selective targeting of MYCN-regulated adrenergic gene expression in NB. Importantly, both CDK9 and CDK2 inhibition are required for maximal effect of CYC065, as CDK9 inhibition alone downregulates MYCN, but fails to induce robust apoptosis, and CDK2 knockout alone is well tolerated in NB cells. Promising results from Mossé and colleagues (50) and our own recent work (20) additionally suggest the ability of proapoptotic agents such as BCL2 inhibitors (e.g., venetoclax), to further enhance effects of transcriptional inhibition. Together, these data establish a compelling therapeutic rationale for rapid clinical evaluation of dual CDK9/2 inhibitors and specifically the oral developmental drug CYC065 in MYCN-driven high-risk NB.

## Methods

### Cell culture.

Cell lines were LGC standards and purchased from the European Collection of Authenticated Cell Cultures (ECACC), ATCC, and Leibniz Institute DSMZ-German Collection of Microorganisms and Cell Cultures and were cultured in RPMI-1640 (MilliporeSigma) or DMEM (MilliporeSigma) as recommended by the suppliers, supplemented with 10% FCS (Gibco; Thermo Fisher Scientific), and maintained at 37°C under 5% CO_2_ in air. All cell lines were verified by STR profiling and routinely tested for mycoplasma contamination.

### Reagents.

CYC202 (seliciclib, *R*-Roscovitine), CCT68127, and CYC065 were provided by Cyclacel Ltd. Cycloheximide (C4859) and ActD (A9415) were purchased from MilliporeSigma and MG132 (1748) from Tocris Bioscience. Temozolomide, flavopiridol, palbociclib, dinaciclib, and SNS-032 were purchased from SelleckChem. BAY 1145372 was purchased from Active Biochem. Compound 3 was provided by Keith Jones (ICR). THZ1 (A8882) was purchased from Stratech. NVP-2 was obtained from Calla Olson, Baylor College of Medicine. THAL-SNS-032 was synthesized in house (27).

### Tumor cell proliferation assays.

Cell proliferation assays were performed as described (51) using the Sulforhodamine B (230162; SRB) assay or using CellTiter-Glo Luminescent Cell Viability Assay (G7571; Promega) and read on a Synergy HT Multi-Mode Microplate Reader (Biotek). GI_50_ values were calculated with PRISM GraphPad, and GI_50_ was defined as the compound concentration at which tumor cell growth was inhibited by 50% compared with the vehicle control. Percentages of viable cells were analyzed using trypan blue exclusion method.

### CDK2 activity detection.

The lentivirus construct of CDK2 sensor was provided by Sabrina L. Spencer (University of Colorado Boulder, Boulder, Colorado, USA). The CDK2 sensor lentiviral particles were produced using second-generation packaging plasmids psPAX2 and pMD2.G obtained from Addgene (a gift from Thomas F. Westbrook, Baylor College of Medicine; Addgene plasmids 1226 and 12259). 293T cells were cultured in DMEM (MilliporeSigma) supplemented with 10% FCS and transfected using TransIT-293 Transfection Reagent (MIR 2704; Mirus). Viral supernatant was collected 48 and 72 hours after infection, filtered through a 0.45 μm low-protein binding filter (HAWP04700; MilliporeSigma), and concentrated with a Lenti-X concentrator (631232; Clontech). Kelly and BE(2)C cells were transduced with concentrated virus in the presence of 8 μg/mL polybrene. After 24 hours, cells were fed with DMEM with 10% FCS. mVenus-positive cells were collected using flow cytometry after 72 hours of infection. mVenus-positive cells were plated in glass-bottom 96-well microplate (655892; Greiner Bio-One). After 24 hours, cells were treated with DMSO, 1 × GI_50_ NVP-2, 2 × GI_50_ NVP-2, or 1 × GI_50_ CYC065 for 2 hours, 4 hours, 6 hours, or 8 hours. Cells were fixed by 4% paraformaldehyde, which was followed by DAPI staining. mVenus fluorescence was imaged by IC200 cytometer (ValaSciences).

### Immunofluorescence.

Immunofluorescence analysis was performed as described (51). Briefly, cells were fixed with ice-cold 4% paraformaldehyde, permeabilized with 0.2% Triton X-100 in PBS, incubated with primary antibody Alexa Fluor 488 or Alexa Fluor 568 secondary antibody, (Life Technologies) and visualized with a Leica DM2500 microscope or quantified with the InCell Analyzer 1000.

### Click-iT RNA imaging kit.

The Click-iT RNA Imaging Assay (C10330, Thermo Fisher) was used to detect newly synthesized RNA. Alkyne-containing nucleoside was incorporated into newly synthesized RNA and detected by an azide containing an FITC fluorescent dye. The assay was conducted according to the manufacturer’s protocol. Briefly, cells were cotreated with 1 mM 5-ethynyl uridine and either DMSO, 1 μM CYC065, or 1 μM CCT68127 for 60 minutes. As a positive control, the general transcription inhibitor ActD (0.5 μg/mL, 60 minutes incubation) was used. The cells were fixed and permeabilized as described above, incubated with Click-iT Reaction Cocktail, and nuclei stained with DAPI. Fluorescence was visualized with a Leica DM2500 microscope and quantified with the InCell Analyzer 1000. Green fluorescence indicated newly synthesized RNA. Nascent RNA was isolated using the Click-iT Nascent RNA Capture Kit (C10365, Thermo Fisher), followed by quantitative PCR (qPCR). Primers for PCR are listed in Table 2.

### FISH.

MYCN FISH (05J50-001, Abbott Molecular) was conducted according to the manufacturer’s protocol. Briefly, Kelly cells were treated with CYC065, fixed with Carnoy’s solution, and codenatured with LSI N-MYC (2q24) Spectrum Orange probe. The melting temperature was set at 73°C (2 minutes) and hybridization temperature at 37°C (overnight). The cells were visualized using a Leica DM2500 microscope. Non–*MYCN*-amplified cells, SK-N-AS and SH-EP, were used as controls.

### Western blot.

Western blot analysis was performed as described (51) using NuPAGE Novex 4% to 12%, and the membranes were exposed using a Fujifilm LAS-4000 Imager, with the Amersham ECL Prime Western Blotting Detection Reagent (GE Healthcare). Antibodies for immunoblots are listed in Table 3.

### shRNA knockdown.

shRNA knockdown experiment was performed using SKNBE cells, and protein was harvested 96 hours after virus transduction and subjected to Western blot analysis. shRNAs for CDK7 and CDK9 (shRNA TRC library) were purchased from MilliporeSigma and are listed in Table 4. SHC002 MISSION pLKO.1-puro Non-Mammalian shRNA Control was used as negative control.

### siRNA knockdown.

siRNA knockdown experiments were performed using Kelly cells. Protein was harvested 96 hours after transfection with siRNA and Dharmafect (Dharmacon) and subjected to Western blot analysis. siRNAs for CDK2 (J003236-12/14) and CDK9 (J003243-14) were purchased from Dharmacon. Nontargeting siRNA control was used as negative control.

### Generation of CDK2 CRISPR cell lines.

To generate Cas9 stable cell lines, Kelly cells were transduced with 1 mL virus and 8 μg/mL Polybrene (Merck Millipore) for 48 hours, selected with 10 μg/mL blasticidin for 10 days, sorted into single cells, and checked for expression of Cas9. Virus was created by transfection of 293T cells with Viral Power Mix (Invitrogen) and a pLenti-Cas9-2A and Blast plasmid (52) (a gift from Jason Moffat, University of Toronto, Toronto, Canada; Addgene 73310).

To generate CDK2 CRISPR stable cell lines, Cas9 stable Kelly cells were transduced with 1 mL virus and 8 μg/mL Polybrene (Merck Millipore) for 48 hours, selected with 1 μg/mL puromycin for 10 days, sorted into single cells, and checked for loss of expression of CDK2. Viruses were created by transfection of 293T cells with Viral Power Mix (Invitrogen) and CDK2 sgRNA (Invitrogen LentiArray Human CRISPR Library CRISPR id 692363). To validate CDK2 knockout, genomic DNA was extracted (Zymo Quick-DNA Microprep; Zymo Research D3020), and sequences of the locus around the putative edit were PCR amplified using target-specific primers (CDK2 sgRNA [CRIPSR ID 692363] forward: 5′-CACCCTGACTACCCAAGAATTAG-3′; reverse: 5′-TGTCAGCCCAGAGAGGATAA-3). The resulting PCR products were purified (DNA clean and concentrator-25, Zymo Research D4033), submitted to Sanger sequencing, and analyzed using the ICE CRISPR Analysis Tool (https://www.synthego.com/products/bioinformatics/crispr-analysis).

### Flow cytometry.

Cells were treated with CYC065 or CCT68127, fixed in cold 70% ethanol, and treated with 40 μg/mL propidium iodide (P4864; MilliporeSigma) and 100 μg/mL RNase A (19101; QIAGEN) before being analyzed using LSR II flow cytometer (BD Biosciences).

### Promoter activity luciferase reporter assay.

IMR-32 cells were transfected with a MYCN promoter Renilla luciferase construct and Cypridina TK control construct (SN0322s; Switchgear Genomics), replated to 96-well plates, and treated with compounds (1 μM) for 6 hours at 48 hours after transfection. Luciferase reading was normalized to the Cypridina TK control signal.

### Tandem ubiquitin binding entity pulldown.

Kelly cells were treated with either DMSO or 1 μM CYC065 for the indicated times, lysed in 50 mM Tris-HCl pH 7.5, 150 mM NaCl, 1 mM EDTA, 1% NP-40, 10% glycerol and 200 μg/mL GST-TUBE2 (UM102; Biosensors, 2BScientific; TUBE indicates tandem ubiquitin binding entity) or in the absence of GST-TUBE2 for control pulldown. Pierce Glutathione Magnetic Beads (88821; Thermo Scientific Fisher) were used to pull down ubiquitinated proteins from cell lysates according to the manufacturer’s instructions. Ubiquitinated proteins were eluted by boiling beads Laemmli buffer and resolved by SDS-PAGE.

### Quantitative RT-PCR and ChIP.

Quantitative RT-PCR and ChIP analysis were performed as described (51). Fluorescence was read using the Step One Plus Real-Time PCR System (Applied Biosystems) with the TaqMan CT/CT program. Analysis was performed using Step One software. TaqMan assays for qPCR are listed in Table 2. Error bars show SD of representative replicate. Primers specific for the APEX gene were as follows: forward: TGAAGCGGGTGTTAGTATGATCT; and reverse: ACCACAAACAACAGAACGAATCT.

### p53 mutational analysis.

Genomic DNA was extracted from cell lines (QIAGEN QIAamp DNA kit). PCR amplification of exons 5 to 9 was performed using the primers shown in Table 5. Products were sequenced with the original PCR primers using the BigDye Terminator Cycle Sequencing Kit and an ABI 3730 Genetic Analyzer (Applied Biosystems). Sequences were analyzed using Mutation Surveyor software, version 3.97 (SoftGenetics).

### RNA-Seq.

RNA extraction was performed by Direct-zol RNA Miniprep Kit (R2050; Zymo Research) with recommended DNase I digestion according to the manufacturer’s instructions. All samples were subjected to quality control on a TapeStation instrument and only RNAs with RNA integrity number (RIN) greater than 8 were used for sequencing. External RNA spike-ins (ERCC, Ambion) were added to total RNA based on cell number. Total RNA and ERCC were subjected to poly(A) selection (E7490; New England BioLabs Inc.). Library preparation of RNA-Seq was performed by using NEBNext Ultra Directional RNA Library Prep Kit for Illumina (E4720L; New England BioLabs Inc.). RNA-Seq libraries were sequenced on a NextSeq 500 (Illumina). NCBI’s Gene Expression Omnibus (GEO) session information for RNA-Seq experiments is in Table 6.

### ChIP using tagmentation.

Antibodies for ChIP using tagmentation (ChIPmentation) were as follows: MYCN (catalog sc-791; Santa Cruz Biotechnology Inc.); H3K27ac (catalog 8173S; Cell Signaling Technology). ChIPmentation was performed as previously described (53). ChIPmentation libraries were sequenced on a NextSeq 500 (Illumina).

### ATAC-Seq analysis.

For each cell line, 50,000 cells were lysed for 10 minutes at 4°C in lysis buffer (10 mM Tris-HCl pH 7.4, 10 mM NaCl, 3 mM MgCl_2_, and 0.1% IGEPAL CA-360). After lysis, pellets were subjected to a transposition reaction (37°C, 60 minutes) using 2× TD buffer and transposase enzyme (Illumina Nextera DNA Preparation Kit, FC-121-1030). The transposition mixture was purified using a QIAGEN MinElute PCR Purification Kit. Library amplification was performed using custom Nextera primers, and the number of total cycles was determined by running a SYBR dye–based qPCR reaction and calculating the cycle number that corresponded to one-fourth the maximum. Amplified libraries were purified using a QIAGEN PCR Purification Kit and sequenced on a single lane of an Illumina NextSeq.

### ChIP-Seq analysis.

MYCN and H3K27ac ChIP-Seq data in the Kelly cell line were obtained from Zeid et al. (13). Briefly, raw reads were aligned using Bowtie2 (version 2.2.1) to build version NCBI37/HG19 (54). Alignments were performed using all default parameters except for –N 1. These criteria preserved only reads that mapped uniquely to the genome with one or fewer mismatches. All analyses were performed using HG19 RefSeq gene annotations.

Normalized read density of a ChIP-Seq data set in any genomic region was calculated using the Bamliquidator read density calculator (https://github.com/BradnerLab/pipeline/wiki/bamliquidator). ChIP-Seq reads aligning to the region were extended by 200 bp, and the density of reads per bp was calculated. The density of reads in each region was normalized to the total number of million mapped reads producing read density in units of reads per million mapped reads per bp (rpm/bp).

Regions of H3K27ac and MYCN enrichment were defined using the model-based analysis of ChIP-Seq (MACS), version 1.4.1, with peak finding algorithm at a *P* value threshold of 1 × 10^–9^ (55). Active promoters were defined as those with an enriched H3K27ac peak in the ±1 kb region flanking the transcription start site (TSS). Active enhancers were defined as regions of H3K27ac outside of this ±1 kb TSS region. For each gene, MYCN promoter and enhancer load were quantified as the cumulative area under the curve MYCN signal in the ±1 kb region (promoter) or within ±50 kb of the TSS (enhancer).

To correlate expression change with MYCN load in Kelly, we first defined active transcribed and expressed genes as those with H3K27ac present in the ±1 kb TSS region and expression in the top 50% of all genes. We ranked these genes by promoter + enhancer MYCN load and binned the top 5000 genes into 5 bins of 1000 genes each. For each bin, average MYCN load was calculated as was the average log_2_ change in mRNA levels after 1 hour CYC065 treatment (Figure 4G). Error bars represent the 95% CIs of the mean as empirically determined by resampling of the data with replacement (10,000 iterations). Sequencing depth of ChIP-Seq experiments is in Table 7.

### ChIPmentation analysis.

MYCN and H3K27ac ChIPmentation data in SH-EP MYCN cells were analyzed using AQUAS TF and the histone ChIP-Seq pipeline (https://github.com/kundajelab/chipseq_pipeline). All analyses were performed using HG19 RefSeq gene annotations. Normalized read density of a ChIPmentation data set in any genomic region was calculated as described in *ChIP-Seq analysis*. Regions of H3K27ac and MYCN enrichment were defined using MACS2 peak-finding algorithm built in AQUAS TF and histone ChIP-Seq pipeline at a *P* value threshold of 1 × 10^–5^. Active promoters and active enhancers were defined as described in *ChIP-Seq analysis*.

### Gene expression analysis.

Total RNA was isolated from cells and tumor tissue using the RNAeasy Plus Minikit (QIAGEN), labeled, and hybridized to GeneChip human or mouse transcriptome expression array (Affymetrix). Results were robust multichip average (RMA) (56) normalized using the limma package from R, and differentially expressed genes were called using a linear model and empirical Bayes statistics from the affy package. For heatmaps showing gene expression changes, genes were filtered based on average expression (log_2_ intensity value >5) and hierarchical clustering using Manhattan distance with complete linkage done in R. Gene set enrichment analyses (GSEA) (57) were performed with the C2 and Hallmark gene set collections from MSigDB, signal2noise metric, and 1000 permutations. mRNA half-lives were taken from Schwanhäusser et al. (58), grouped in short (<5 hours) and long (>18 hours) half-life, and the log_2_ fold change in mRNA expression upon CYC065 treatment was illustrated as box plot. Boxes represent the first and third quartile; the middle line reflects the median; and whiskers extend to ×1.5 interquartile range. Outliers are shown as dots. *P* values were calculated using 2-tailed Wilcoxon’s rank sum test. The log_2_ fold change of median of ADRN CRC or MES CRC upon the DMSO group was represented using a heatmap.

### RNA-Seq analysis of SH-EP MYCN cell lines.

Reads were aligned to the human reference genome hg19/GRCh37 using HISAT2 with parameter --no-unal. Gene expression values (fragments per kilobase per million reads [FPKM]) were computed using Cufflinks, version 2.2.1, with library type fr-firststrand. Cell number–normalized FPKM were calculated based on ERCC RNA Spike-In Mix (Thermo Fisher Scientific). ADRN and MES gene sets were taken from van Groningen et al. (12), and the log_2_ fold change in mRNA expression upon the SH-EP or DMSO group was illustrated as a box plot. Boxes represent the first and third quartile; the middle line reflects the median; and whiskers extend to ×1.5 interquartile range. *P* values were calculated with 2-tailed Welch’s *t* test. GSEA (57) was performed with the C2 and Hallmark gene set collections from MSigDB, Signal2Noise metric, and 1000 permutations. The log_2_ fold change of median of ADRN CRC or MES CRC upon the DMSO group was represented using a heatmap.

### In vivo efficacy of CYC065 in human xenograft models and GEM mice.

Female CrTac:NCr-*Foxn1^nu^* athymic nude mice (Taconic) (6 weeks of age) were injected with either Kelly (5 × 10^6^ cells), SK-N-AS (5 × 10^6^ cells), or H128 (5 × 10^6^ cells) subcutaneously in 1 flank and allowed to establish. Mice bearing NB xenografts with a mean diameter of 5 mm were treated with 75 mg/kg/d CYC065 or vehicle (saline) dosed orally, using a 5 days on, 2 days off schedule for up to 3 weeks. Tumor volumes were measured by Vernier caliper across 2 perpendicular diameters, and volumes were calculated according to the following formula: *V* = 4/3π [(*d1* + *d2*)/4]^3^ where *d1* and *d2* are the 2 perpendicular diameters. Transgenic TH*-MYCN* or TH-*ALK^F1174L^/TH-MYCN* mice were genotyped to detect the presence of human MYCN or ALK transgene (59). Male or female mice with palpable tumors (30–50 days old) were treated with 50 mg/kg of CYC065, CCT68127, vehicle (saline), freshly prepared 6 mg/kg temozolomide, or a combination of either 50 mg/kg of CYC065 or 50 mg/kg of CCT68127 with freshly prepared 6 mg/kg temozolomide for 2 consecutive weeks. CYC065 or CCT68127 were dosed using a 5 days on, 2 days off schedule. Mice were allowed access to sterile food and water ad libitum.

### MRI.

Changes in tumor volume in the TH-*MYCN* or TH-*ALK^F1174L^/TH-MYCN* mice were quantified using MRI on a 7T horizontal bore MicroImaging system (Bruker Instruments) using a 3 cm birdcage coil. Anatomical T_2_-weighted coronal images were acquired through the mouse abdomen, from which tumor volumes were determined using segmentation from regions of interest (ROI) drawn on each tumor-containing slice. The T_1_ and ADC, 2 functional MRI parameters, were also measured (38). At trial end, tumors were dissected and fixed with 4% paraformaldehyde or snap-frozen in liquid nitrogen for further analysis.

### Pathology.

Tissue sections were stained with H&E or specific antibodies. Immunohistochemistry was performed using standard methods. Briefly, 5 μm sections were stained with antibodies, including heat-induced epitope retrieval of specimens using citrate buffer (pH 6) or EDTA buffer, and scored by a consultant histopathologist.

Tumor or spleen tissue was homogenized using T-PER buffer (Thermo Fisher Scientific) containing proteinase inhibitor (Roche) and a cocktail of phosphatase inhibitors (Santa Cruz Biotechnology Inc.). Protein (30 mg) was denatured in lithium dodecyl sulfate sample buffer (Invitrogen), separated on precast 4%–12% Bis-Tris gels (Invitrogen), and transferred to nitrocellulose membranes for Western blotting. Immunoblots were recorded electronically on a Fujifilm LAS-4000 scanner.

### Data availability.

ChIP-Seq and RNA-Seq data have been deposited in the GEO database (GSE107126, GSE80151, GSE128330, GSE145068).

### Statistics.

Data were visualized and statistical analyses performed using GraphPad Prism (version 6; GraphPad Software Inc.) or the R statistical package. For each group of data, estimate variation was taken into account and is indicated in each figure as SD or SEM. If single data are presented, these data are representative of biological or technical triplicates, as indicated. Statistical analyses between groups with comparable variance were performed using 2-tailed unpaired Student’s *t* test unless otherwise indicated. Pearson’s tests were used to identify correlations among variables. Significance for all statistical tests is shown in figures or legends. *P* < 0.05 was considered significant. No samples or animals were excluded from analysis, and group sizes were determined by power analyses using data previously shown (38, 51). Animals were randomly assigned to groups. Studies were not conducted blinded, with the exception of all histopathological scoring.

### Study approval.

All experimental protocols were monitored and approved by the ICR Animal Welfare and Ethical Review Body, in compliance with guidelines specified by the UK Home Office Animals (Scientific Procedures) Act 1986 and the United Kingdom National Cancer Research Institute Guidelines for the Welfare of Animals in Cancer Research (60).

## Author contributions

EP, TL, YJ, CYL, and LC conceived and designed the study. EP, TL, YJ, RZ, SW, CK, A Hakkert, KB, ZU, KT, A Hallsworth, G Box, MEE, MPL, YS, GL, EC, BMC, MV, ADHB, HW, NT, GSA, RC, G Boysen, MWR, G Barone, AF, RB, PAC, JDB, NSG, JB, SPR, SAE, DZ, PW, JEB, JM, IV, ME, CYL, and LC conducted experiments and analyzed and interpreted data. EP, TL, YJ, PW, ME, CYL, and LC wrote the manuscript. All authors read and approved the final manuscript.

## Supplementary Material

Supplemental data

## Figures and Tables

**Table 3:**
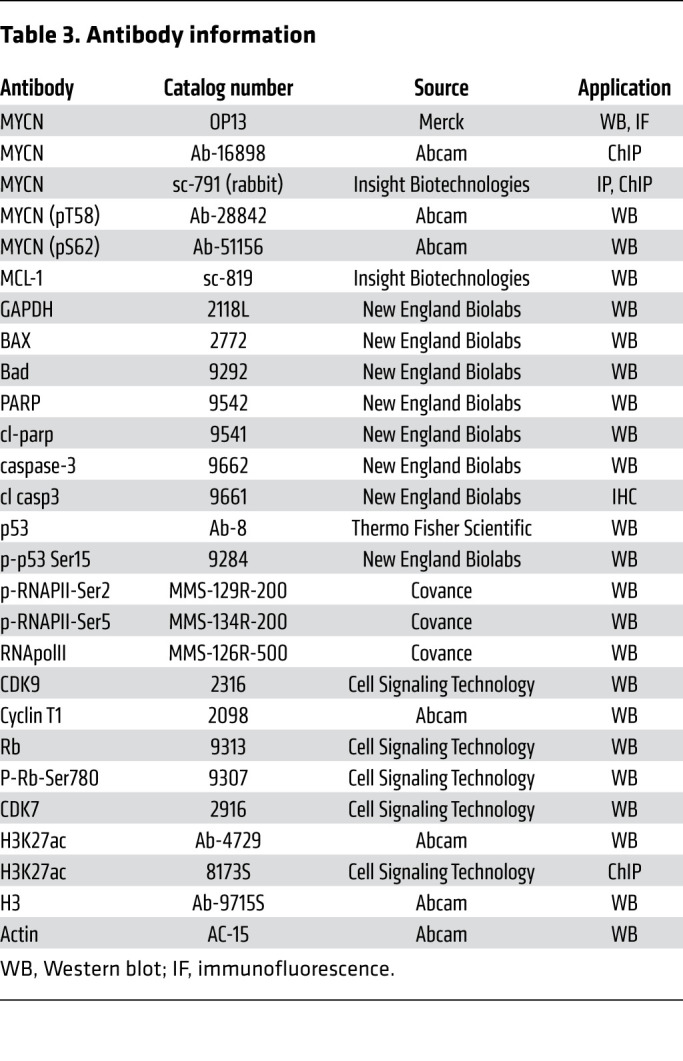
Antibody information

**Table 6:**
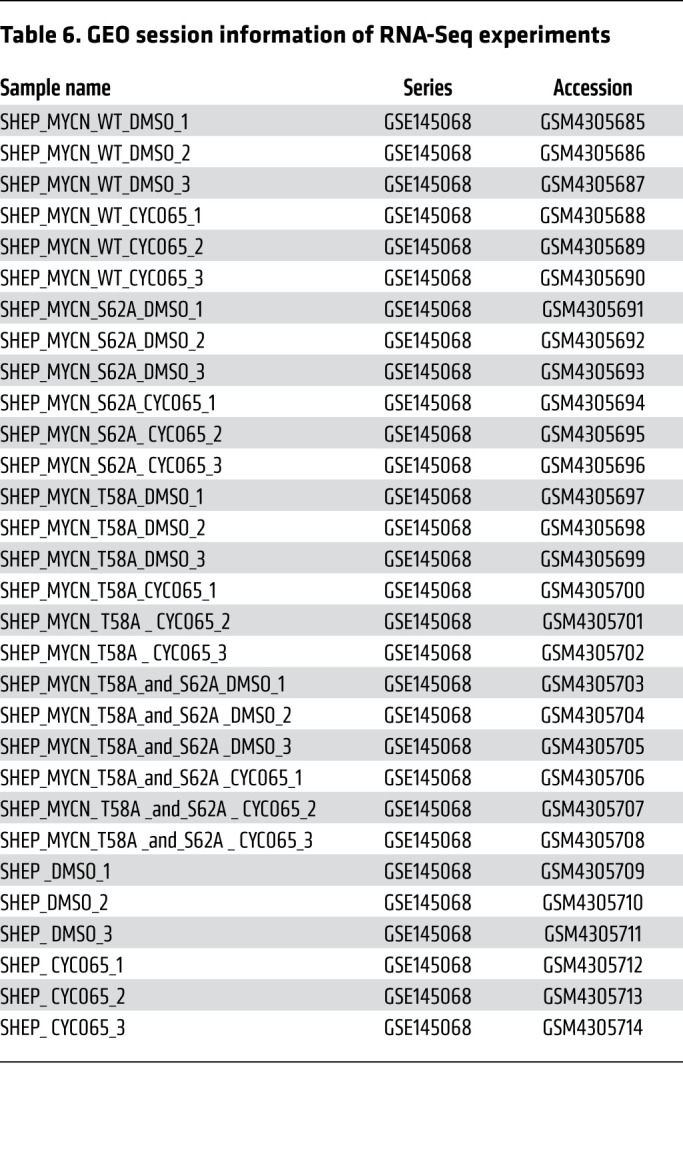
GEO session information of RNA-Seq experiments

**Table 4:**
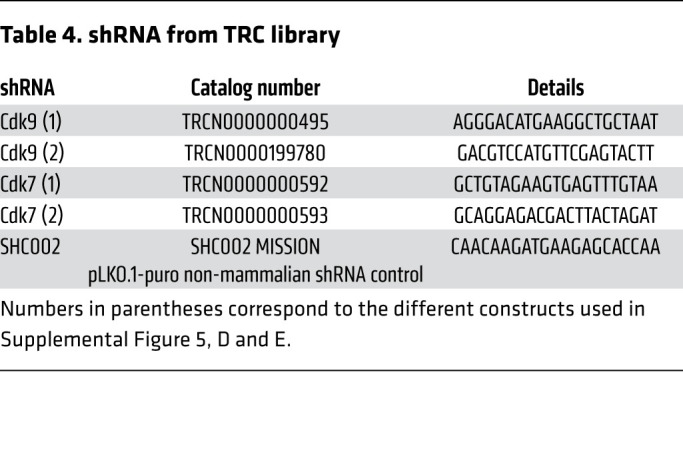
shRNA from TRC library

**Table 7:**
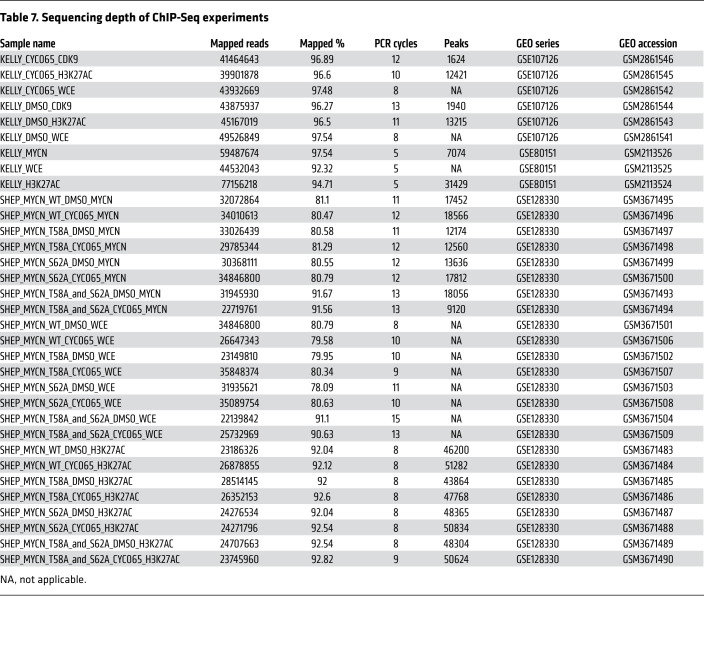
Sequencing depth of ChIP-Seq experiments

**Table 1:**
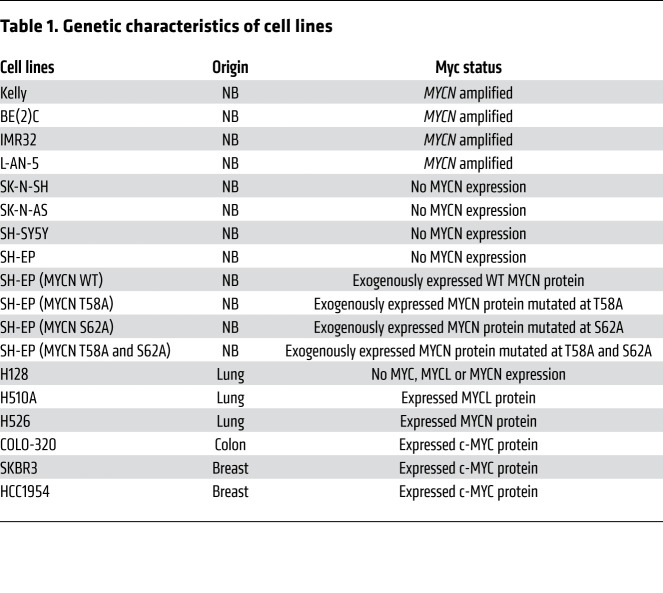
Genetic characteristics of cell lines

**Table 5:**
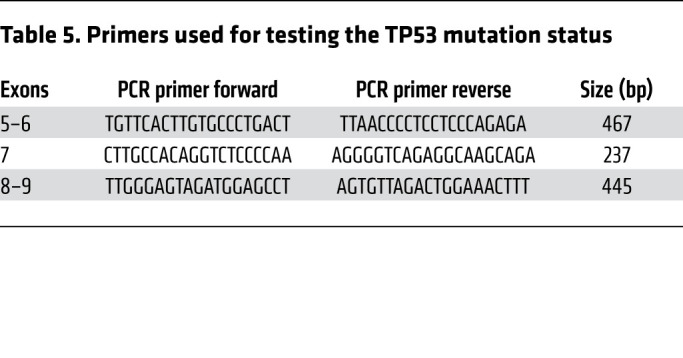
Primers used for testing the TP53 mutation status

**Table 2:**
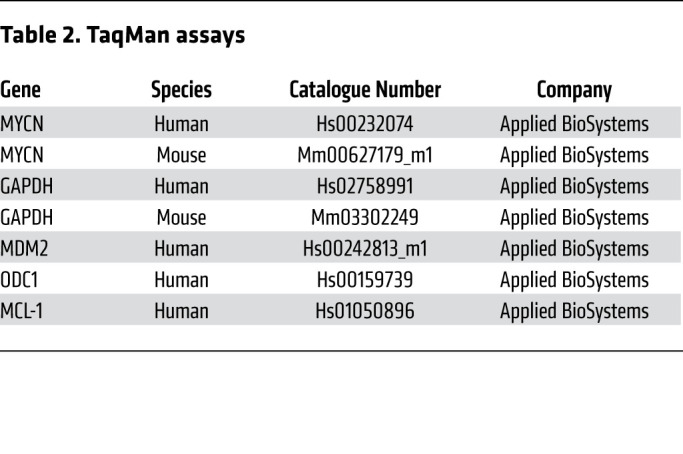
TaqMan assays

## Version 1 10/05/2020

Electronic publication

## Version 2 11/02/2020

Print issue publication
