# Supplementary material for: Orally bioavailable CDK9/2 inhibitor shows mechanism-based therapeutic potential in MYCN-driven neuroblastoma
Source: J Clin Invest. 2020 Oct 5;130(11):5875–92. doi: 10.1172/JCI134132 (PMC7598076; doi:10.1172/JCI134132)
Supplement: Supplemental data [file jci-130-134132-s155.pdf]

Supplemental Figure 1

A

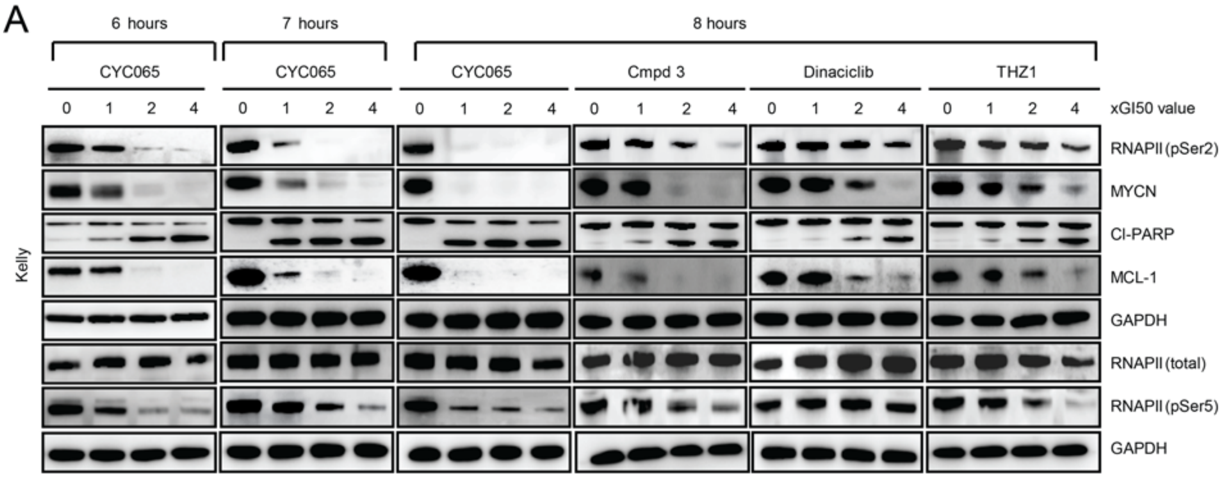

Inhibitor	Selectivity (nM) CDK2	CDK7	CDK9	Others
CYC065	8	612	14	CDK5
CCT68127	10	520	9	CDK5
Compound 3	580		14	
Dinaciclib	1		4	CDK1(3), CDK5(1)
THZ1		3.2		
SNS-032	62	38	4	
Flavopiridol	405	514	11	CDK1(27), CDK4(132)
BAY1143572			13	
Palbociclib				CDK4(11), CDK6(16)

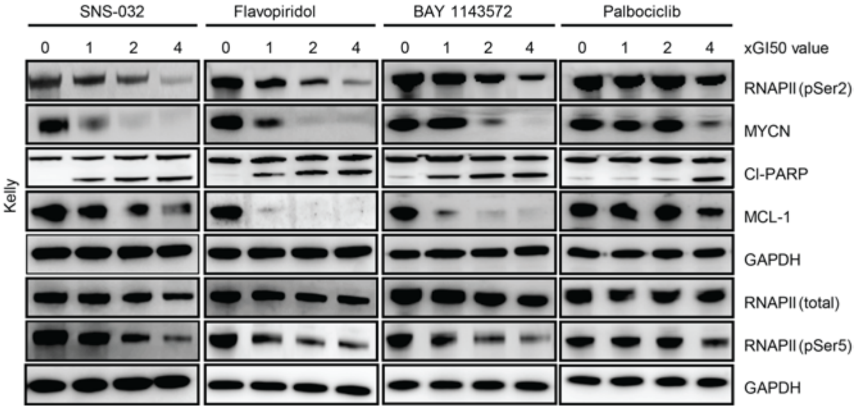

Supplemental Figure 1: *MYCN*-amplified neuroblastoma is sensitive to CDK9 inhibitors.

A, Immunoblots depict effects of treatment with CDK inhibitors at the indicated time and concentrations in Kelly cells (n=1-3). Table showing reported selectivity of the CDK inhibitors(16, 26, 27, 50).

Supplemental Figure 2

A

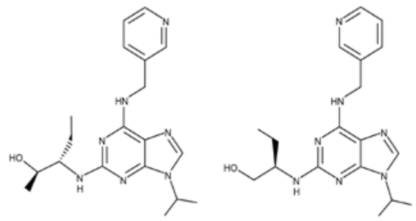

B

Inhibitor	GI50 (72 hrs) nM	% Viable cells
CYC202	6250	NT
CCT68127	853	52
CYC065	430	47
Compound 3	80	76
Dinaciclib	3	78
THZ1	30	83
SNS - 032	498	65
Flavopiridol	60	87
BAY1143572	501	83
Palbociclib	4957	81

C

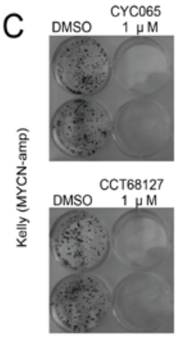

D

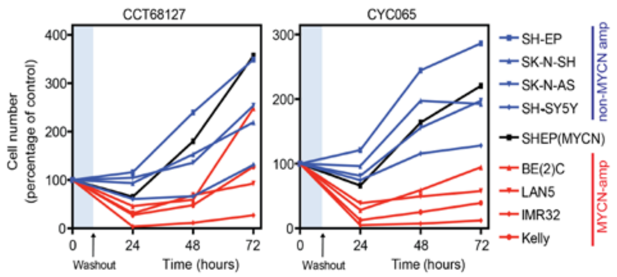

E

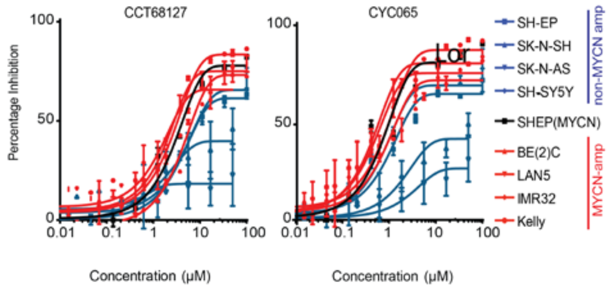

F

		DMSO	CCT68127	CYC065
Kelly	G0/G1	46.9	47.4	45.4
	S	27.9	22.3	27.1
	G2M	23.2	17.1	12
	Sub G1	1.1	12.5	15.1
BE(2)C	G0/G1	45	36.2	31.3
	S	25.3	22.1	24.7
	G2M	27.5	27.9	18.1
	Sub G1	1.4	10.7	23.2
SH-EP	G0/G1	61.3	55.5	53.5
	S	14.8	18.2	21.6
	G2M	21.2	21.2	19.1
	Sub G1	2.2	4.5	4.9
SK-N-AS	G0/G1	45.4	42.7	47.3
	S	24.6	21.5	20.9
	G2M	28.3	33.8	29.4
	Sub G1	0.5	0.8	0.9

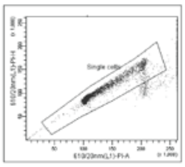

G

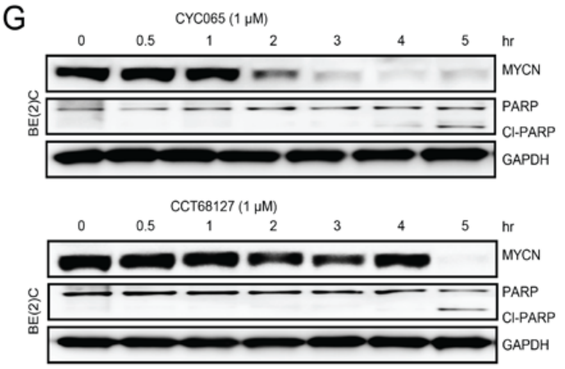

H

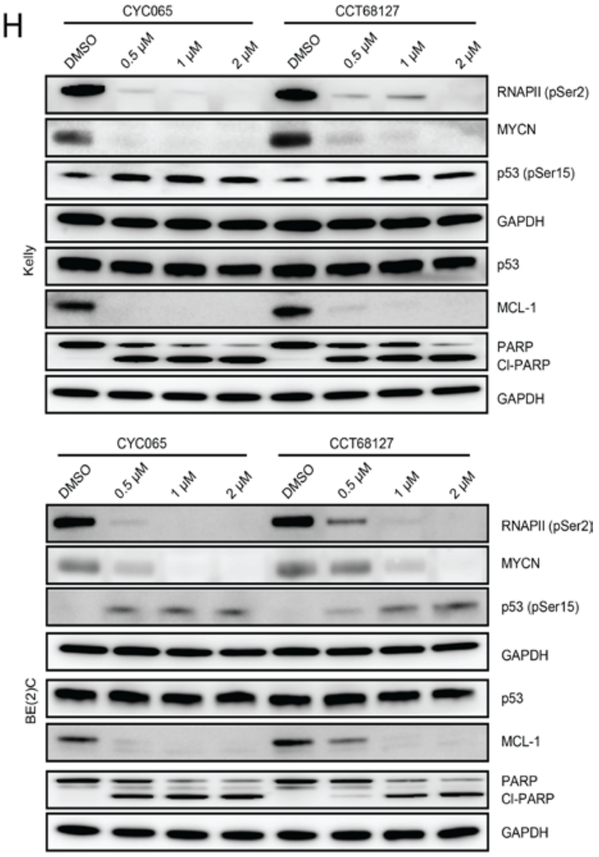

I

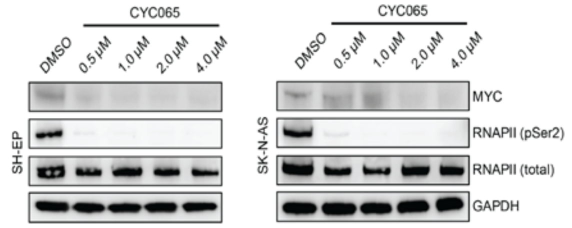

J

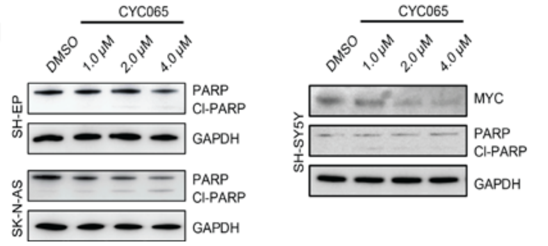

Supplemental Figure 2: CYC065 and CCT68127 target MYCN-driven neuroblastoma and induce apoptosis.

A, Structures of CCT68127 and seliciclib (CYC202).

B, GI50 of CCT68127, CYC065, seliciclib (CYC202) and other CDK inhibitors in Kelly NB cells. Cells were treated continuously with each compound for 72 hr and GI50 values (nM) were calculated after 72 hr using an SRB assay (n= 3).

C, Crystal violet-stained culture dishes of Kelly NB cells after treatment with CYC065 or CCT68127 (1 µM, 8hr) or vehicle (DMSO), replated and replaced with normal growth media for 7 days (n=2).

D, Proliferation of NB cells over 72hr quantified using a Cell-titerGlo assay. Cells were treated with CYC065 (1 µM) and CCT68127 (2 µM) for 8hr, washed off and replaced with normal growth medium (n=3).

E, GI50 curve of Figure 1b (n= 3).

F, Flow cytometry analysis showing cell cycle phases of *MYCN*-amplified (Kelly, BE(2)C) and non-*MYCN*-amplified (SH-EP, SK-N-AS) cells in response to CYC065 or CCT68127 (1 µM; 8hr) as indicated in Fig 1e. A pulse geometry gate PI-H x PI-A was used to gate out the doublets and debris (n= 2).

G, BE(2)C cells were treated with CYC065 or CCT68127 at 1 µM for 0.5-5hr. Immunoblots depict expression of MYCN and cleaved PARP (n= 2).

H, Immunoblots depict expression of MYCN, MCL-1, PARP, p53, p-p53 at Ser15 and GAPDH in Kelly and BE(2)C cells following treatment with CYC065 or CCT68127 (0.5-2 µM; 8hr) (n= 2).

I, Immunoblots showing expression of MYC, RNAPII Ser2P and GAPDH in SH-EP and SK-N-AS cells following treatment with CYC065 (0.5-4 µM; 8hr) (n= 2).

J, Immunoblots depict expression of cleaved PARP and GAPDH in SH-EP, SK-N-AS and SH-SY5Y cells following treatment with CYC065 (1-4 µM; 8hr) (n= 2).

Supplemental Figure 3

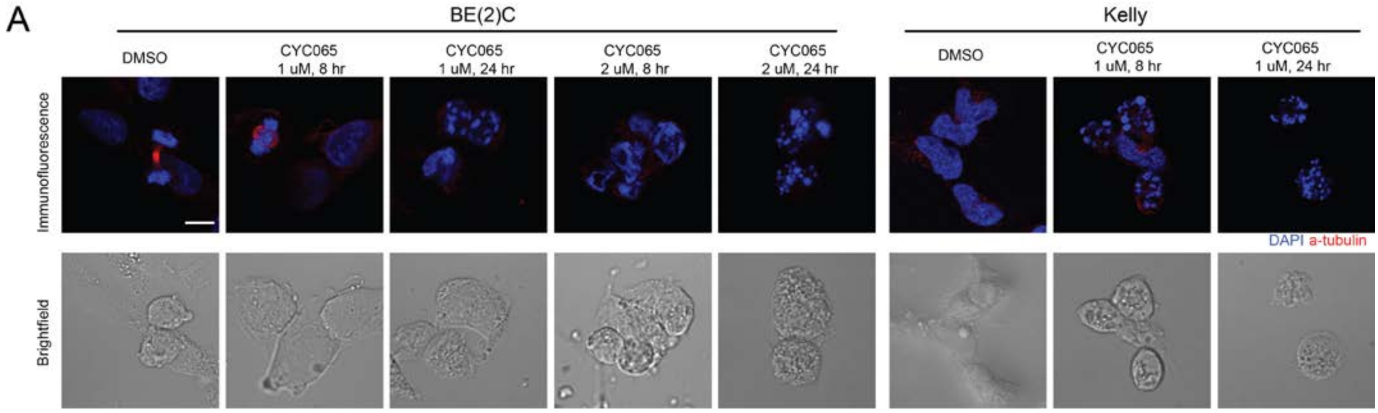

Supplemental Figure 3: CYC065 does not induce anaphase catastrophe in MYCN-driven neuroblastoma.

A, Immunofluorescence and brightfield images documenting morphological changes and staining with α-tubulin (red) and nuclei Dapi (blue) in BE(2)C and Kelly cells after treatment with CYC065 (1-2 µM, 8-24hr). DMSO control cells shows two spindle poles. Multipolar anaphases (multiple spindle poles) was not detected in CYC065 treated cells. Scale bars = 5 µm (n= 3).

Supplemental Figure 4

A

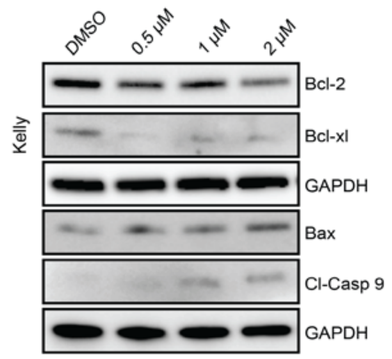

B

Cell line	TP53 status	Protein description
BE(2)C	Mutated	P.C135F
Kelly	Mutated	P.P177T
IMR32	Wild type	
L-AN-5	Wild type	

C

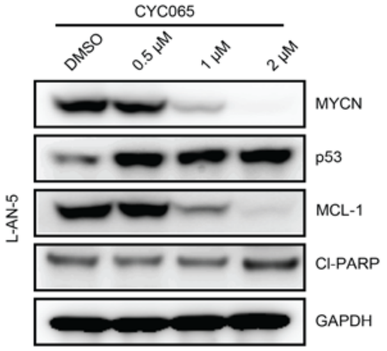

D

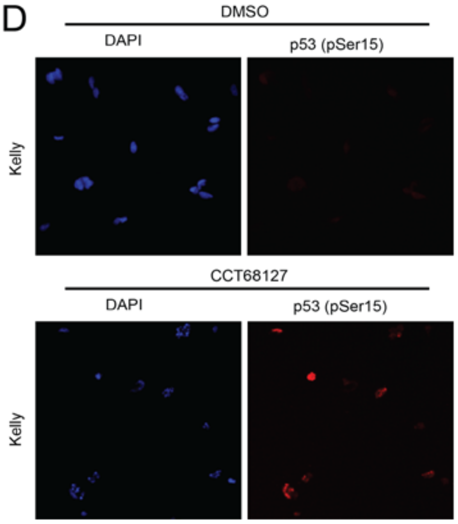

E

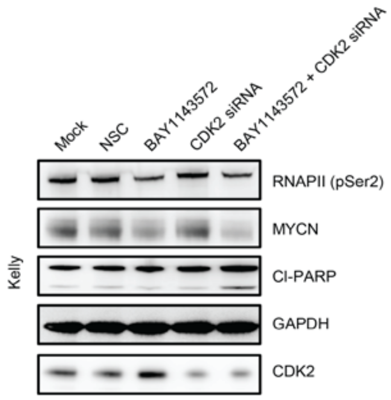

F

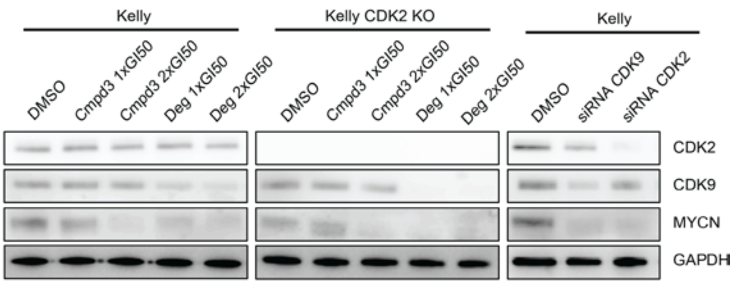

G

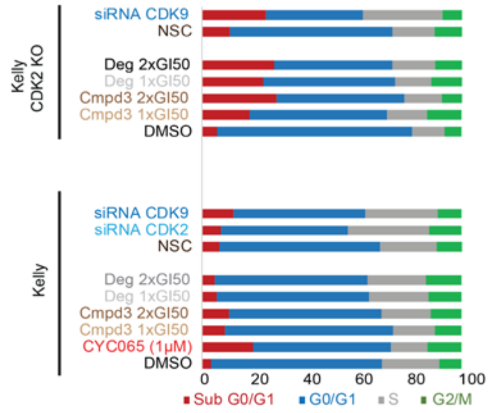

Supplemental Figure 4: CDK9 and CDK2 inhibition induces TP53 mediated apoptosis.

A, Immunoblots showing expression of the indicated proteins and GAPDH in Kelly cells following treatment with CYC065 (0.5-2 µM, 6hr).

B, TP53 mutation status of the tested cells.

C, Immunoblots depict expression of p53, p-p53 at Ser15, MYCN, MCL-1, PARP and GAPDH in p53 native L-AN-5 cells following treatment with CYC065 or CCT68127 at the indicated concentrations for 6hr.

D, Immunofluorescence showing p-p53 at Ser15 (red) in Kelly cells after treatment with CCT68127 for 6hr.

E, Immunoblots depict expression of MYCN, CDK2 and PARP cleavage in Kelly cells after treatment with BAY1143572 (GI50, 6hr), CDK2 siRNA (48hr) or both (pretreatment with CDK2 siRNA for 42hr before treatment with BAY1143572 for a further 6hr).

F, Immunoblots showing expression of MYCN after treatment with Compound 3 (Cmpd3) and CDK9 degrader THAL-SNS-032 (1-2xGI50, 8hr), genetic knockdown of CDK9 or CDK2 by siRNA (48hr) and genetic knockout of CDK2 by CRISPR in Kelly cells.

G, Kelly cell cycle profile after genetic knockdown of CDK9 or CDK2 by siRNA (48hr), genetic knockout of CDK2 by CRISPR and/or treatment with Compound 3 (Cmpd3) and CDK9 degrader THAL-SNS-032 (1-2xGI50, 8hr). GI50 of THAL-SNS-032 is 40 nM.

Supplemental Figure 5

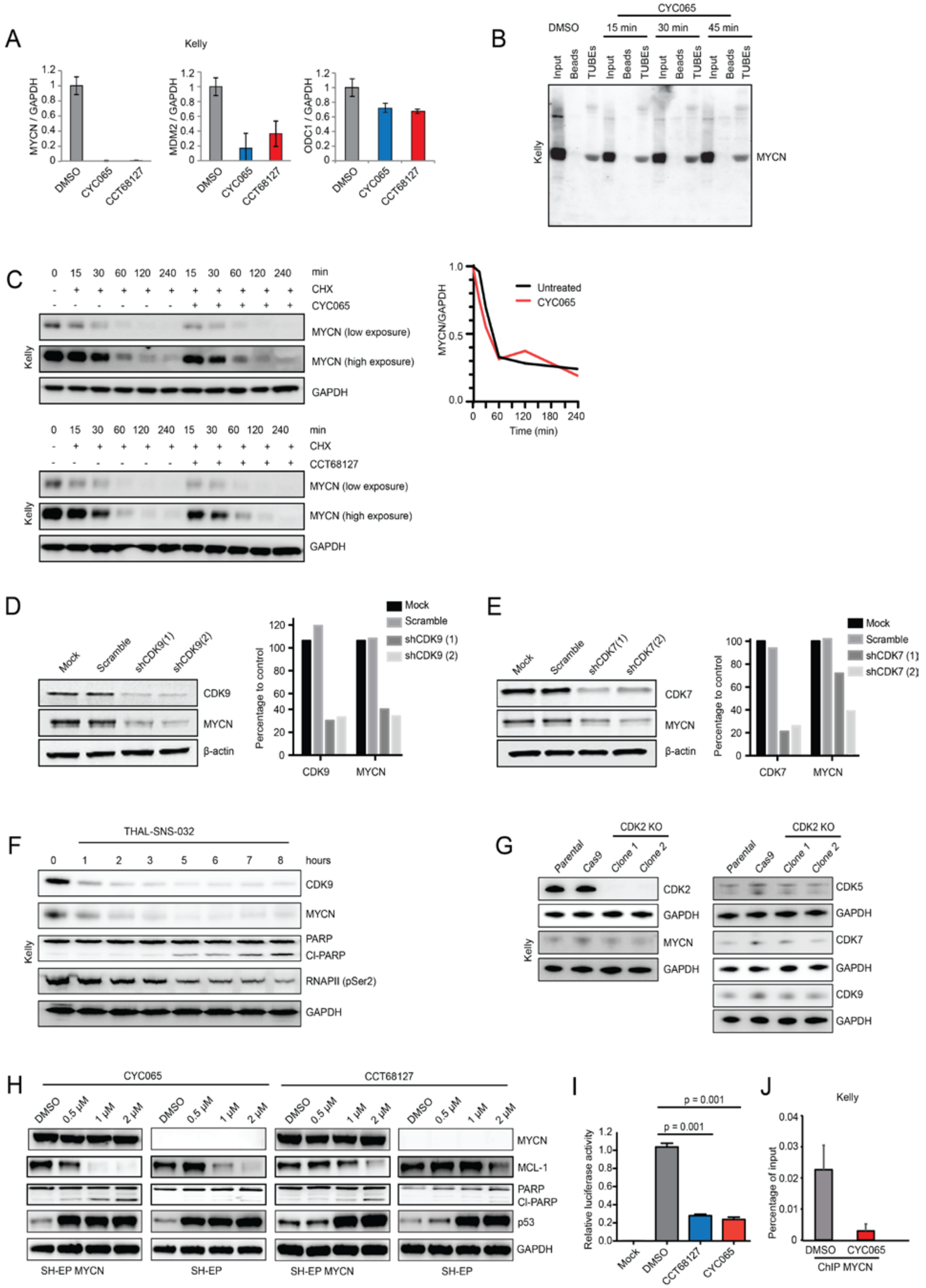

Supplemental Figure 5: Effects of inhibition of CDK9, CDK7 and CDK2 on MYCN protein.

A, Quantitative RT-PCR showing MYCN genes and MYCN target genes, MDM2 and ODC1, after treatment with CYC065 or CCT68127 (1 µM, 8hr) in Kelly NB cells (+/- S.D. of two independent experiments; Significance was calculated using two-tailed unpaired Student's t-test).

B, Tandem Ubiquitin Binding Entity (TUBE) pulldown assay showing no change in ubiquitinated MYCN protein after treatment with CYC065 (1 µM) at the indicated time.

C, BE(2)C cells were treated with CYC065 (1 µM) or CCT68127 (1 µM) and cycloheximide (25 µg/ml), harvested at the indicated time points, and immunoblotted for MYCN protein.

D, Immunoblots and graph showing expression of CDK9 and MYCN proteins when CDK9 is down-regulated by shRNA.

E, Immunoblots and graph showing expression of CDK7 and MYCN proteins when CDK7 is down-regulated by shRNA.

F, Immunoblots showing effect of CDK9 degrader THAL-SNS-032 (0.1 µM, 1-8hr) on CDK9, cleaved PARP, phosphorylated RNAPII Ser2 and MYCN protein levels

G, Immunoblots showing genetic knockout of CDK2 in Kelly cells.

H, Immunoblot showing the effects of CYC065 or CCT68127 (6hr) in native SH-EP cells that lack the native transcriptional machinery of MYCN and SH-EP MYCN WT cells with exogenously-expressed MYCN WT.

I, Cells were transfected with a MYCN promoter Renilla luciferase construct and Cypridina TK control construct, and treated with compounds (1 µM) for 6hr at 48hr post transfection. Luciferase reading was normalized to the Cypridina TK control signal (+/- S.D. of two independent experiments).

J, Results of ChIP assays using MYCN at a genomic region surrounding the E box of the APEX gene after treatment with CYC065 (1 µM, 1hr). (+/- S.D. of three independent experiments).

Supplemental Figure 6

A

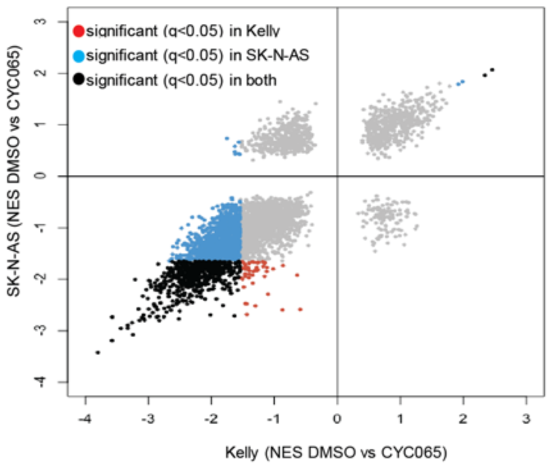

B

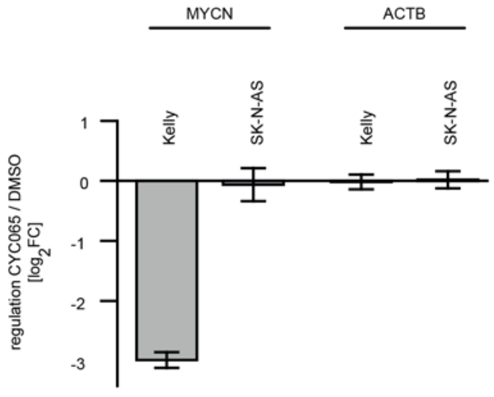

C

gene set	Kelly NES	Kelly q-value	SK-N-AS NES	SK-N-AS q-value
Reactome: Generic transcription pathway	-3.8	<1e-4	-3.4	<1e-4
Reactome: RNA POL II transcription	-2.7	<1e-4	-2.0	2.0e-3
Reactome: RNA POLI, RNA POLIII and mitochondrial transcription	-2.5	<1e-4	-2.8	<1e-4
PID: TRKR pathway	-1.7	<2e-2	-0.7	<1e0

D

gene set	TH-MYCN NES	TH-MYCN q-value
Reactome: Transcription	-2.7	<1e-4
Reactome: RNA POLI, RNA POLIII and mitochondrial transcription	-2.5	<1e-4
Reactome: RNA POL II transcription	-2.5	<1e-4
Rhodes: Undifferentiated cancer	-2.4	<1e-4

E

Gene set	Kelly		BE(2)C		SK-N-AS		SH-SY5Y		TH-MYCN tumors	
	NES	FDR	NES	FDR	NES	FDR	NES	FDR	NES	FDR
MYC targets V2	-2.46	<1.0e-4	-1.95	<1.0e-4	-1.85	1.72e-3	2.19	<1.0e-4	-1.95	5.20e-4
MYC targets V1	-1.46	2.53e-2	-1.38	6.88e-2	-1.00	5.32e-1	-0.69	1.00e0	-2.90	<1.0e-4
Yu: MYC targets up	-2.31	8.41e-6	-1.58	8.36e-2	-1.45	1.33e-1	-1.63	3.36e-2	-2.73	<1.0e-4
Schlosser: MYC targets and serum response up	-2.23	8.47e-5	-1.68	5.31e-2	-1.78	1.99e-2	-1.62	3.70e-2	-1.66	3.17e-2
Ben-Porath: MYC targets with E-box	-1.93	3.05e-3	-1.62	6.91e-2	-1.57	7.66e-2	-1.47	9.10e-2	-0.79	9.62e-1
Acosta: Proliferation independent MYC targets up	-1.91	3.84e-3	-1.55	9.33e-2	-0.59	1.00e-0	-1.25	2.57e-1	-1.03	5.61e-1
Dang: Regulated by MYC up	-1.89	4.24e-3	-1.25	3.01e-1	-1.00	6.91e-1	-1.30	2.02e-1	-2.33	1.68e-4

Supplemental Figure 6: Gene expression changes upon CYC065 treatment.

A, The xy plot shows overall changes in GSEA after CYC065 treatment (1 µM, 1hr) in Kelly and SK-N-AS cells. Each dot represents one gene set from the MSigDB C2 collection. Significant gene sets with an FDR q-value <0.05 are highlighted.

B, Expression changes of MYCN upon CYC065 treatment in Kelly and SK-N-AS cells used in Affymetrix gene expression array as demonstrated in **c.** Plotted are log_2 fold changes and 95% confidence intervals of 3 biological replicates. B-actin (ACTB) as a not-regulated control.

C, Summary of GSE analyses demonstrate repression of genes after CYC065 treatment in Kelly and SK-N-AS cells.

D, Summary of GSE analyses in CYC065 treated TH-*MYCN* mice.

E, Gene set enrichment analysis in *MYCN*-amplified (Kelly, BE(2)C), *MYCN* non-amplified (SK-N-AS, SH-SY5Y) NB cell lines and tumors from TH-*MYCN* mice after treatment with CYC065. Selected MYC gene sets from the C2 collection of the MSigDB are shown. Gene sets with a non-significant Benjamini-Höchberg-corrected p-value (FDR <0.25) are highlighted in red.

Supplemental Figure 7

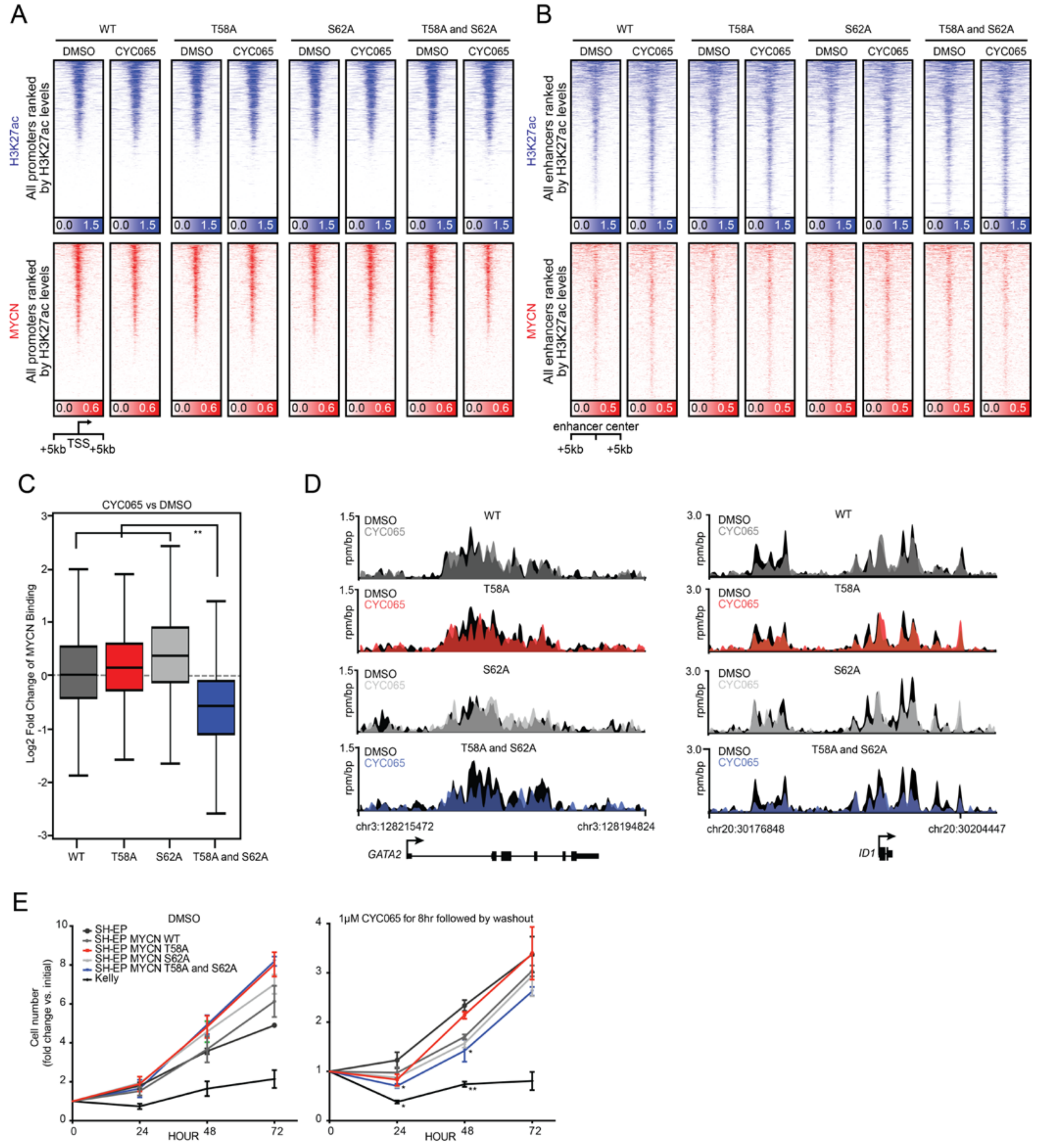

Supplemental Figure 7: CYC065 fails to alter global H3K27ac and MYCN occupancy in SH-EP MYCN lines.

A, Heatmaps of H3K27ac (blue) and MYCN (red) occupancy at all promoters (+/- CYC065, 1 µM, 6hr) treatment ranked by SH-EP MYCN WT DMSO H3K27ac signal.

B, Heatmaps of H3K27ac (blue) and MYCN (red) occupancy at all enhancers (+/- CYC065, 1 µM, 6hr) treatment ranked by SH-EP MYCN WT DMSO H3K27ac signal.

C, Box plot showing the $\log_2$ fold change of genome-wide MYCN occupancy with CYC065 (1 µM, 6hr). Bold line represents median, box represents the interquartile range (IQR), whiskers represent 1.5 times the IQR, and outliers are not shown. Significance is indicated (Welch's two-tailed t test and Benjamini and Hochberg correction): $^{**}P < 1x10^{-8}$ and $FDR < 1x10^{-8}$.

D, Gene tracks MYCN (+/- CYC065, 1 µM, 6hr) occupancy at individual loci. ChIP-Seq occupancy is provided in units of reads per million per base pair (rpm/bp).

E, Proliferation of NB cells over 72hr quantified using a Cell-titerGlo assay. Cells were treated with DMSO or 1 µM CYC065 for 8hr. DMSO or CYC065 treatment were washed off by PBS twice and replaced with normal growth medium (+/- S.D. of three independent experiments). For 24hr, SH-EP vs SH-EP MYCN (T58A), SH-EP vs SH-EP MYCN (T58A and S62A) and SH-EP vs Kelly were statistically significant. For 48hr, SH-EP vs SH-EP MYCN (WT), SH-EP vs SH-EP MYCN (S62A), SH-EP vs SH-EP MYCN (T58A and S62A) and SH-EP vs Kelly were statistically significant. Significance is indicated (two-tailed student t test and Benjamini and Hochberg correction): $^{*}P < 0.05$ and $FDR < 0.05$, $^{**}P < 1x10^{-4}$ and $FDR < 1x10^{-3}$.

Supplemental Figure 8

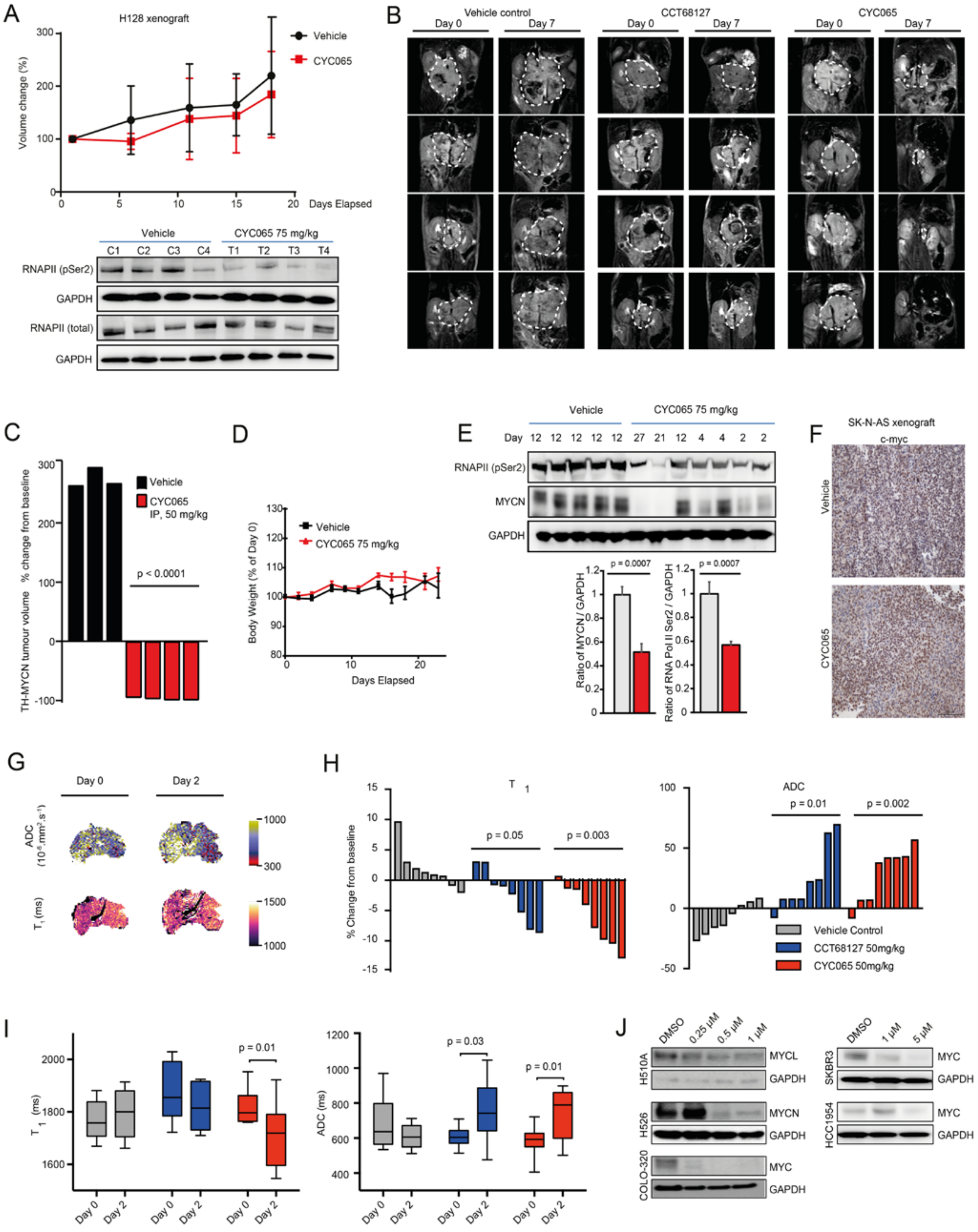

Supplemental Figure 8: Molecular and non-invasive MRI biomarker of response to CYC065 in vivo*.*

A, Effects of CYC065 on the growth and survival of H-128 (non Myc-driven) lung xenografts in mice. Data are expressed as the mean relative tumor volumes (compared with tumor size at the start of treatment) +/- S.E.M. (n=6 vehicle, n=6 (75 mg/kg CYC065). Mice were treated in a 'five days on, two days off' schedule.

B, Coronal T_2-weighted MRI images of the abdomen of four representative tumor-bearing TH-*MYCN* mice prior to and 7 days following a 'five days on, two days off' schedule with CYC065 (50mg/kg), CCT68127 (50mg/kg) or vehicle (--- tumor).

C, Waterfall plot documenting relative changes in tumor volume in the TH-*MYCN* GEM model following seven-day treatment with 50mg/kg CYC065, (p<0.001) in a 'five days on, two days off' schedule. Route of administration: IP (Significance was calculated using two-tailed unpaired Student's t-test).

D, Body weights for treated and control TH-*MYCN* mice.

E, Representative Kelly xenografts harvested at the indicated time for immunoblot analysis for MYCN, p-RNAPII-Ser2 and GAPDH. Graph showing level of MYCN and p- RNAPII-Ser2 after treatment (Significance was calculated using two-tailed unpaired Student's t-test).

F, Representative SK-N-AS xenografts harvested at the end of the trial from Figure 6b for immunohistochemical analysis for c-MYC. Scale bar: 100µm.

G, Parametric functional MRI maps showing the change in the apparent diffusion coefficient (ADC) and tumor spin lattice relaxation time T_1 values 24hr after treatment with vehicle control.

H, Waterfall plot showing relative changes in median native T_1 and ADC in the TH-*MYCN* GEM model 24hr following treatment with 50mg/kg CCT68127, 50mg/kg CYC065 or vehicle. (Significance was calculated using two-tailed unpaired Student's t-test with a 5% level of significance).

I, Change in median native T_1 and ADC values prior to (D0) and 24hr (D2) after treatment with 50mg/kg CYC065 or vehicle. (Significance was calculated using two-tailed paired Student's t-test with a 5% level of significance).

J, Immunoblots showing the effects of 8hr treatment of CYC065 on MYC family members in lung (H510A and H526), colon (COLO-320) and breast (SKBR3 and HCC1954) cancer cell lines.
